# Current population structure and pathogenicity patterns of *Ascochyta rabiei* in Australia

**DOI:** 10.1099/mgen.0.000627

**Published:** 2021-07-20

**Authors:** Ido Bar, Prabhakaran Thanjavur Sambasivam, Jenny Davidson, Lina M. Farfan-Caceres, Robert C. Lee, Kristy Hobson, Kevin Moore, Rebecca Ford

**Affiliations:** ^1^​ Centre for Planetary Health and Food Security, School of Environment and Science, Griffith University, QLD 4111, Australia; ^2^​ South Australian Research and Development Institute, Hartley Grove, Urrbrae SA 5064, Australia; ^3^​ Centre for Crop and Disease Management, School of Molecular and Life Sciences, Curtin University, Bentley, WA 6102, Australia; ^4^​ Department of Primary Industries Tamworth Agricultural Institute, Calala, NSW 2340, Australia

**Keywords:** Ascochyta, chickpea, plant pathogen, population genetics

## Abstract

Ascochyta blight disease, caused by the necrotrophic fungus *Ascochyta rabiei*, is a major biotic constraint to chickpea production in Australia and worldwide. Detailed knowledge of the structure of the pathogen population and its potential to adapt to our farming practices is key to informing optimal management of the disease. This includes understanding the molecular diversity among isolates and the frequency and distribution of the isolates that have adapted to overcome host resistance across agroecologically distinct regions. Thanks to continuous monitoring efforts over the past 6 years, a comprehensive collection of *A. rabiei* isolates was collated from the major Australian chickpea production regions. To determine the molecular structure of the entire population, representative isolates from each collection year and growing region have been genetically characterized using a DArTseq genotyping-by-sequencing approach. The genotyped isolates were further phenotyped to determine their pathogenicity levels against a differential set of chickpea cultivars and genotype-phenotype associations were inferred. Overall, the Australian *A. rabiei* population displayed a far lower genetic diversity (average Nei’s gene diversity of 0.047) than detected in other populations worldwide. This may be explained by the presence of a single mating-type in Australia, MAT1-2, limiting its reproduction to a clonal mode. Despite the low detected molecular diversity, clonal selection appears to have given rise to a subset of adapted isolates that are highly pathogenic on commonly employed resistance sources, and that are occurring at an increasing frequency. Among these, a cluster of genetically similar isolates was identified, with a higher proportion of highly aggressive isolates than in the general population. The discovery of distinct genetic clusters associated with high and low isolate pathogenicity forms the foundation for the development of a molecular pathotyping tool for the Australian *A. rabiei* population. Application of such a tool, along with continuous monitoring of the genetic structure of the population will provide crucial information for the screening of breeding material and integrated disease management packages.

## Data Summary

An online dataset containing all supporting genotyping and phenotyping data and the code required to reproduce the results, summary tables and plots found in this publication, is publicly available at Zenodo via the following links: https://zenodo.org/record/4311477; DOI: 10.5281/zenodo.4311477 [1].

**Impact Statement**

This article is the first published study that utilized a genotyping-by-sequencing approach, providing a much higher resolution data than was previously available on the genetic diversity of the Australian population of a major chickpea pathogen, *Ascochyta rabiei*. The studied population is unique in its isolation and the presence of only a single mating type, which dictates a clonal reproduction mode and limits the genetic diversity of the population. This study demonstrates that despite this limited genetic diversity, the pathogen is capable of adapting and overcoming host resistance sources, as evidenced by the discovery of novel genetic clusters associated with highly pathogenic isolates, posing a major risk to the industry. The study further identifies interesting evolutionary patterns suggesting that *A. rabiei* exhibits local micro-evolution within the broader growing region, in addition to evidence of cross-region gene flow, likely driven by human activities.

These findings and further advances in the understanding of the pathogen populations have significant implications for development of accurate and informed disease management strategies at a regional and national scale to assist Australian scientists, breeders, farmers and government agencies in managing and reducing the chickpea Ascochyta blight risk and allowing sustainable production of chickpea.

## Introduction

With the ongoing growth of the human population, agricultural production systems are facing the increasing challenge of providing sustainable food sources to ensure food security for all [2]. Particular emphasis is given to increasing the production of high nutrition plant-based protein sources, such as legumes, which in addition can fix nitrogen in the soil, making them ideal for crop rotation practices [3]. Chickpea is one of the main crops of the legume family, known for its high fibre, protein and vitamin content [4].

Australia is a major chickpea producer, with the bulk of the Australian crop primarily grown for export to the Indian sub-continent. With peak production exceeding 2 million tonnes in 2016, that was valued at over AU$2 billion, Australia is one of the world’s largest chickpea ‘cash crop’ exporters [5]. Chickpea is grown in Australia in several unique agroecological zones, mainly in the east of the continent, from central Queensland (QLD) through New South Wales (NSW), Victoria (VIC) and South Australia (SA), with some minor production in western and northern Western Australia (WA). Despite substantial expansion in Australian chickpea production over the past decade, from 230 kilo-tonnes (kt) in 2006–2007 to 2000 kt in 2016–2017, yield remains threatened by a range of biotic (pests, weeds, pathogen-borne diseases) and abiotic (changes in climate conditions causing extreme temperatures, drought, frost or floods) factors.

One of the major biotic constraints to chickpea production is Ascochyta Blight, a disease caused by the necrotrophic fungal pathogen *Ascochyta rabiei*. Without proper informed disease management, which includes adoption of resistant cultivars, clean seed, pre-emptive fungicide spraying and rotation of crops, epidemics can cause up to 100 % yield loss (KM, personal communications). This translates to substantial economic losses to growers [6]. The increased demand and record-high profits in Australia in 2016 led many farmers to overlook crop rotation and clean-seed practices, which might have contributed to a rise in the number of highly aggressive *A. rabiei* isolates, as was observed in the following years [7]. These isolates are capable of causing severe disease symptoms, such as stem lesions and breakage on the most resistant chickpea cultivars [6, 8]. The mechanisms underlying this rapid change in pathogenicity are not yet understood.

To effectively manage the risk of Ascochyta blight outbreaks and to choose the most suitable cultivars for a growing region, as well as to advise on disease epidemiology, prevention and management strategies, it is essential to understand the pathogen population structure within each growing region across Australia. This will help to assess the adaptation potential of *A. rabiei* to overcome host resistance and chemical management strategies implemented by growers. Furthermore, the potential identification of fungal adaptation patterns unique to a growing region will assist in predicting future fungal evolution and epidemiology in response to particular farming practices and/or climatic factor trends.

In this study, a genotyping-by-sequencing (GBS) approach was employed to identify single nucleotide polymorphism (SNP) markers among a representative collection of Australian *A. rabiei* isolates. The SNP data was then used to infer population structure within and among growing regions and to identify SNPs, and hence potential genes, associated with high levels of pathogenicity towards commonly grown chickpea cultivars.

## Methods

### *A. rabiei* isolates

The Australian *A. rabiei* isolates used in this study were collected between 2013 and 2018 inclusive, as part of a 6 year monitoring program funded by the Grain Research and Development Corporation (GRDC project ID UM00052; https://grdc.com.au/research/projects/project?id=2023). The isolates were collected from commercial chickpea crops and National Variety Trial (NVT) sites in a quasi-hierarchical manner, where infected material was collected from the four corners and one central location within each field that had been reported as infected. At NVT sites, infected material was collected from as many host genotypes as possible, one sample from each genotype row at each location. Field collection was performed by trained senior plant pathologists from Australia’s Agriculture and Primary Industries Research agencies in each state (South Australian Research and Development Institute, Agriculture Victoria and WA/QLD/NSW Departments of Primary Industries).

All of the isolates used in this study were confirmed as *A. rabiei* through disease symptomology and species-specific molecular testing [9]. Another two isolates P2 and P4 [10], provided by Professor Diego Rubiales (Institute for Sustainable Agriculture, Cordoba, Spain) were derived from the International Center for Agricultural Research in the Dry Areas (ICARDA) collection. These two isolates were confirmed as MAT 1–1 using the PCR assay developed by Barve *et al*. [9] and were used as outgroups, while the mating type for all of the Australian isolates were assessed using the same assay (see Supplementary Material available in the online version of this article for assay details and results). Passport data for the isolates is provided in Table S1, including date of collection, source (geographic and host cultivar), mating type, simple sequence repeat (SSR) haplotypes as determined by Mehmood *et al.* [8] and additional classification derived from this study. Disease scoring assays were performed in a controlled-environment growth room using a mini-dome assay to classify the isolates into pathogenicity groups, as described by Chen *et al.* [11] and refined by Mehmood *et al.* [8] and Sambasivam *et al.* [12]. Specifically, isolates were assessed for their ability to cause disease symptoms on a differential set of chickpea cultivars, ranging from resistant to susceptible in their reaction to Ascochyta blight (ICC3996, Genesis090, PBA HatTrick and Kyabra, respectively) [12]. The disease reaction on each host differential was ranked as low, moderate or high based on a 1–9 qualitative scale [11, 13] and the overall pathogenicity group (PG) was assigned by the additive effect, with specific criteria as detailed in Table 1.

**Table 1.** An example of pathogenicity group assignment of *A. rabiei* isolates based on disease responses on the differential host set

Isolate	Year	State	ICC3996	Genesis090	HatTrick	Kyabra	Patho. group*
17CUR005	2017	VIC	High	High	High	High	5
FT13092-1	2013	SA	Moderate	High	High	High	4
F15027	2015	SA	Moderate	Moderate	High	High	3
16CUR014	2016	VIC	Low	Moderate	High	High	2
AS18084	2018	VIC	Moderate	Low	High	High	2
TR6400	2014	NSW	Low	Low	High	High	1
13RUP002	2013	VIC	Low	Low	Low	High	0

*Classification into pathogenicity groups (PG) was determined by the following criteria: low disease response on all hosts (except Kyabra, which is the susceptible check) – PG0; high on PBA HatTrick and low on ICC3996 and Genesis090 – PG1; high on PBA HatTrick and a combination of low and moderate on ICC3996 and Genesis090 – PG2; high on PBA HatTrick and Moderate on both ICC3996 and Genesis090 – PG3; high on PBA HatTrick and Genesis090 and moderate on ICC3996 – PG4; and high on ICC3996, Genesis090 and PBA HatTrick – PG5.

### Genotyping-by-Sequencing

GBS was performed on the two outgroup isolates sourced from ICARDA and a subset of 279 isolates that was selected from the Australian *A. rabiei* collection described above, representing the range of collection years, geographical regions, host cultivars and pathogenicity groups (see details in Table S1). Numbers of isolates assessed from each of the classification criteria varied and were dependent on the occurrence of an epidemic within a region and within a year (Table 2). DNA was extracted from the selected 279 isolates using a modified cetyl trimethylammonium bromide (CTAB) extraction method [14]. The DNA samples were genotyped using DArTseq (Diversity Array Technologies P/L, Canberra, Australia) [15, 16].

**Table 2.** Number of Australian *A. rabiei* isolates used for DArTseq genotyping by year and state∗

Year	NSW	QLD	SA	VIC	WA	Total
2013	0	0	23	3	0	26
2014	24	0	4	8	0	36
2015	0	0	9	7	0	16
2016	8	5	12	5	13	43
2017	17	16	14	15	11	73
2018	16	1	24	20	24	85
Total	65	22	86	58	48	**279**

*Australian states are abbreviated in the table as follows: NSW, New South Wales; QLD, Queensland; SA, South Australia; VIC, Victoria; WA, Western Australia.

### Population genetics analysis

Variant tables produced by DArTseq were imported into the R language and environment for statistical computing as *adegenet* (v2.1.1) genlight objects [17] using the *dartR* (v1.1.11) package [17–19]. The SNP dataset was filtered to remove loci that did not show any variability (monomorphic) or did not pass the filtering criteria of reproducibility (DArTseq RepAvg score > 0.95) or coverage (read depth > 5). Also, loci and individuals with low genotype call rates (> 20 % missing data) were removed. When multiple SNPs were identified on a single tag (locus), a single representative SNP was selected from each tag using the ‘best’ approach implemented in *dartR*. The locus was removed if it contained five or more SNPs.

The filtered SNP set was used to calculate a matrix of pairwise genetic distances between each pair of isolates, which was used for principal components (PC) analysis with the *ade4* (v1.7.13) and *adegenet* R packages [19–22]. This was achieved by retaining principal components that explained at least 60 % of the cumulative variance. Summarized genetic similarity between populations were assessed from the SNP set by calculating the Euclidean distances and presented as a neighbour-joining phylogenetic tree, as implemented by the gl.tree.nj() function in *dartR* [18].

### Clonality and population analysis

To assess the level of clonality in the population, the retained SNP markers were used to define multilocus genotypes (MLGs), representing a unique combination of alleles in each isolate, while allowing a minimal degree of freedom using a distance threshold ~1, to allow for genotyping errors and missing values. Isolates that shared the same MLG were considered to be clones of the same lineage and were collapsed together for downstream analysis of the *A. rabiei* population structure. To visualize the genetic relationships between MLGs, a minimum spanning network (MSN) was constructed, based on a Euclidean distance dissimilarity matrix between each pair of MLGs. Population diversity metrics, including the Shannon–Weiner Diversity index; Stoddard and Taylor’s index; Simpson’s index; corrected Simpson’s index (Λ∙N/(N-1)); genotypic evenness index; Nei’s gene diversity (expected heterozygosity); and clonal fraction (1-MLG/N), were calculated using *poppr* (v2.8.3) based on the MLG analysis [23, 24]. The analyses were performed following the best practices for population genetic analysis of clonal fungal pathogens, as specified by Grünwald *et al.* [25].

### Isolate relatedness

To determine the pairwise genetic distances between isolates, the Euclidean distance between each pair of isolates was calculated based on the allele frequencies of the filtered SNP set [26]. The distance matrix was used by *pheatmap* (v1.0.12) [27] to cluster the isolates and plot a heatmap and a dendrogram to visually represent isolate relatedness and identify patterns associated with origin and pathogenicity. The proportion of MLGs comprising isolates from multiple clusters was calculated to assess the robustness of the MLG and cluster assignment.

Isolates were further classified into ‘low’ and ‘high’ pathogenicity if they were assigned to pathogenicity groups 0–2 or 3–5, respectively. This separated between isolates that were able to cause moderate and severe disease symptoms on the more resistant host cultivars Genesis 090 and ICC3996. This classification was converted into a binary format (0,1) and used as the ‘response’ in a generalized linear mixed-effect model (GLMM) to determine the effect of the assigned cluster, using a binomial model, as implemented by the *lme4* (v1.1.21) R package [28, 29]. The odds ratio was calculated to reflect the differences in proportions of ‘high’ and ‘low’ pathogenicity isolates between the clusters and the Tukey method was applied on the log of the odds ratio scale to determine statistical significance.

### Variant association with pathogenicity

To identify putative allelic variants that contributed to pathogenicity, a genome-wide association study (GWAS) approach was employed using the filtered SNP data of the genotyped population. The analysis was performed with *SNPassoc* (v1.9.2) R package [30], assuming a ‘log-additive’ model and a significance threshold that was adjusted to reduce the false discovery rate in the case of multiple testing (*q-*value ≤ 0.05) [31].

Annotation of the genes at each significantly associated SNP locus was performed based on the reference genome and gene models of *A. rabiei* isolate ArME14 (GCA_004011695.1) [32]. Additional functional annotation of these loci was performed using homology searches using blast against the NCBI non-redundant protein database (nr) and by InterProScan against the InterPro conglomerate databases [33, 34]. Effector prediction of the variant-associated sequences was performed by EffectorP (v1.0/2.0) [35, 36].

### Supporting data

An online dataset containing all supporting genotyping and phenotyping data and the code required to reproduce the results, summary tables and plots found in this publication, is publicly available at Zenodo via the following links: https://zenodo.org/record/4311477; DOI: 10.5281/zenodo.4311477 [1].

## Results

**Fig. 1.**
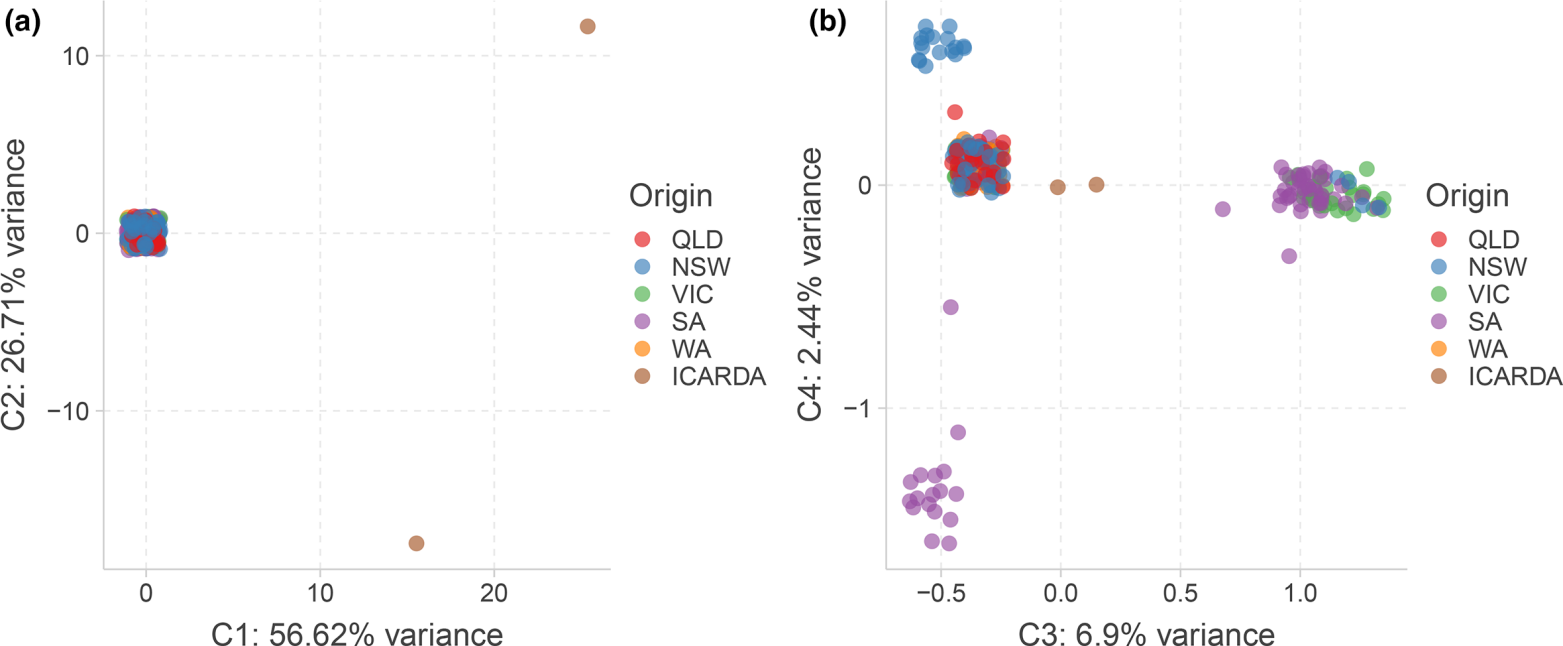
Principal component analysis representing the genetic distance between all isolates. The first two principal components are presented in (a) and components 3 and 4 are presented in (b). Isolates are coloured by their origin: QLD, Queensland; NSW, New South Wales; VIC, Victoria; SA, South Australia; WA, Western Australia; and ICARDA, International Centre for Agricultural Research in the Dry Areas (Middle East).

All of the Australian isolates were confirmed as mating type MAT 1–2 via PCR (see Fig. S1 in Supplementary Material). While the main focus of this study was the genetic diversity of the Australian *A. rabiei* population, we included two MAT 1–1 isolates from a Northern Hemisphere collection (ICARDA), distinct from the isolated population from Australia, in our genotyping analysis. A total of 1474 SNPs were detected from all the samples and was used for PC analysis. The analysis revealed that most of the variance (approximately 82 %) in the genetic distance between the isolates was derived from the two outgroup isolates sourced from ICARDA (Fig. 1a). The Australian isolates showed very low genetic diversity and overlay each other in Fig. 1(a). The two outgroup isolates displayed a significantly greater genetic diversity compared to the Australian isolates and to one another. The genetic distance within the Australian isolates was visible only when focusing on the third and fourth PC and showed a major cluster of very similar isolates from all regions, and three distinct clusters of isolates separated by their state of origin (Fig. 1b). Nei’s gene diversity for the P2 and P4 isolates from ICARDA was 0.313, and that of the Australian and ICARDA isolates collectively as a single population was 0.102. Inclusion of P2 and P4 isolates from the ICARDA collection in a phylogenetic tree illustrated the potential extent of *A. rabiei* genetic diversity globally and emphasized the limited genetic diversity in the Australian population (Fig. S2).

### The Australian *A. rabiei* population structure inferred from DArTseq

After removal of the two outgroup isolates and focusing on the sampled population of Australian *A. rabiei* isolates (*n*=279), only 229 SNPs were polymorphic. Additional quality filtration removed one locus with a high rate of missing data (> 20 %), seven loci with low reproducibility scores (< 0.95) and an additional nine secondary SNPs that shared the same sequence tags (loci) and were predicted to be linked to existing markers. Finally, 212 high-confidence polymorphic SNPs were retained for subsequent analyses of the Australian population.

The retained SNPs were used to define multilocus genotypes (MLGs), representing a unique combination of alleles for each isolate, in a similar way that the SSR haplotypes were previously reported and assessed for Australian *A. rabiei* [8]. For this, 185 contracted MLGs were determined to represent the genotypic variability among the sampled 279 isolates, with 94 isolates considered as clones.

The Australian *A. rabiei* population contained very low genetic diversity across the years sampled with an average expected heterozygosity (Hexp) of 0.016 and minor seasonal fluctuations ranging from a low of Hexp=0.012 in 2015 to Hexp=0.021 in 2013. The observed higher diversity in 2013 was supported by a higher number of expected MLGs and low clonal fraction (Table 3). An examination of the population diversity within each state revealed an extremely low diversity among isolates originating from WA (Hexp=0.006 and CF=0.458), indicating that close to half of the 48 isolates (within our collection) were considered as genetic clones of existing MLGs. Higher diversity was found among isolates collected in other states, with similar Hexp of approximately 0.016±0.001, and particularly in QLD with a CF=0 (Table 3).

**Table 3.** *A. rabiei* population diversity metrics derived from DArTseq data within each collection year (2013–2018) and geographic region (state)

Pop	N	MLG	eMLG	SE	H	G	Hexp	E.5	Lambda	Lambda*	CF
2013	26	25	15.631	0.483	3.205	24.143	0.021	0.979	0.959	0.997	0.038
2014	36	30	14.356	1.065	3.283	21.600	0.016	0.803	0.954	0.981	0.167
2015	16	12	12.000	0.000	2.361	9.143	0.012	0.848	0.891	0.950	0.250
2016	43	33	13.823	1.239	3.318	20.775	0.014	0.743	0.952	0.975	0.233
2017	73	54	14.136	1.257	3.756	29.442	0.015	0.681	0.966	0.979	0.260
2018	85	57	13.747	1.353	3.742	26.661	0.013	0.623	0.962	0.974	0.329
QLD	22	22	22.000	0.000	3.091	22.000	0.015	1.000	0.955	1.000	0.000
NSW	65	51	19.640	1.335	3.791	35.504	0.016	0.797	0.972	0.987	0.215
VIC	58	45	19.012	1.434	3.625	27.574	0.015	0.727	0.964	0.981	0.224
SA	86	64	18.824	1.571	3.883	28.667	0.018	0.582	0.965	0.976	0.256
WA	48	26	14.233	1.607	2.745	7.629	0.006	0.455	0.869	0.887	0.458
**Total**	**279**	**185**	**19.191**	**1.633**	**4.738**	**42.236**	**0.016**	**0.364**	**0.976**	**0.980**	0.337

MLG – the number of multilocus genotypes found in the specified population; eMLG – the expected number of MLG at the lowest common sample size; SE – the standard error for the rarefaction analysis; H – Shannon–Weiner diversity index; G – Stoddard and Taylor’s index; Hexp – Nei’s gene diversity (expected heterozygosity); Lambda – Simpson’s index; E.5 – genotypic evenness; Lambda* – corrected Simpson’s index: Λ∙N/(N-1); CF – clonal fraction: (1-MLG/N). Highly clonal populations in each subset are highlighted in orange, while high-diversity ones are highlighted in green, based on the clonal fraction values. Australian states are abbreviated in the table as follows: NSW, New South Wales; QLD, Queensland; SA, South Australia; VIC, Victoria; WA, Western Australia.

**Fig. 2.**
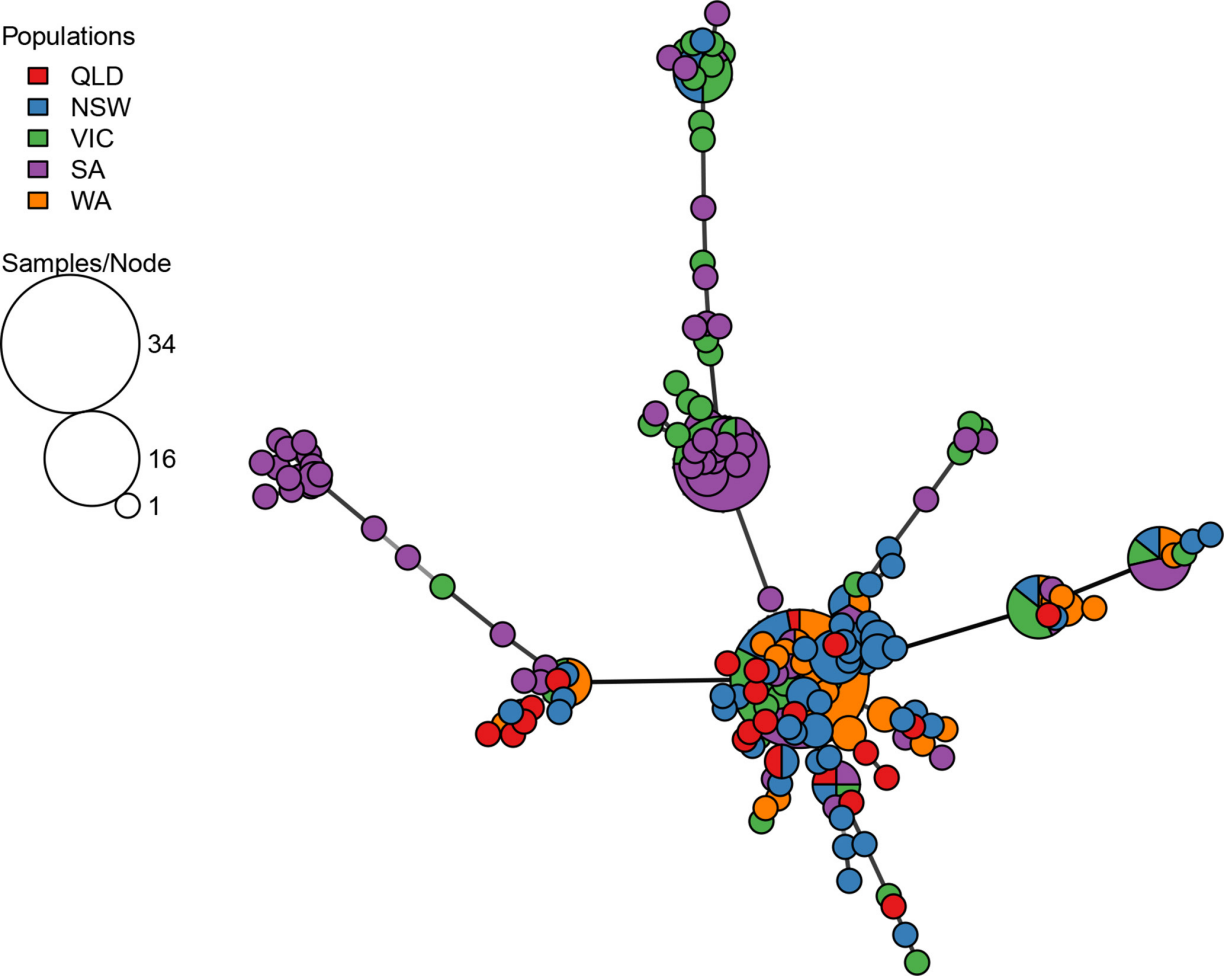
Minimum spanning network diagram of multilocus genotypes by state. Where node size represents the number of isolates identified in each MLG, and the length of the branches between the nodes represents the genetic distance. Node colours represent the origin of the isolates in the MLG.

To visualize the genetic relationships detected between MLGs, a minimum spanning network (MSN) was constructed, highlighting the number of isolates in each MLG and their state of origin (Fig. 2).

**Fig. 3.**
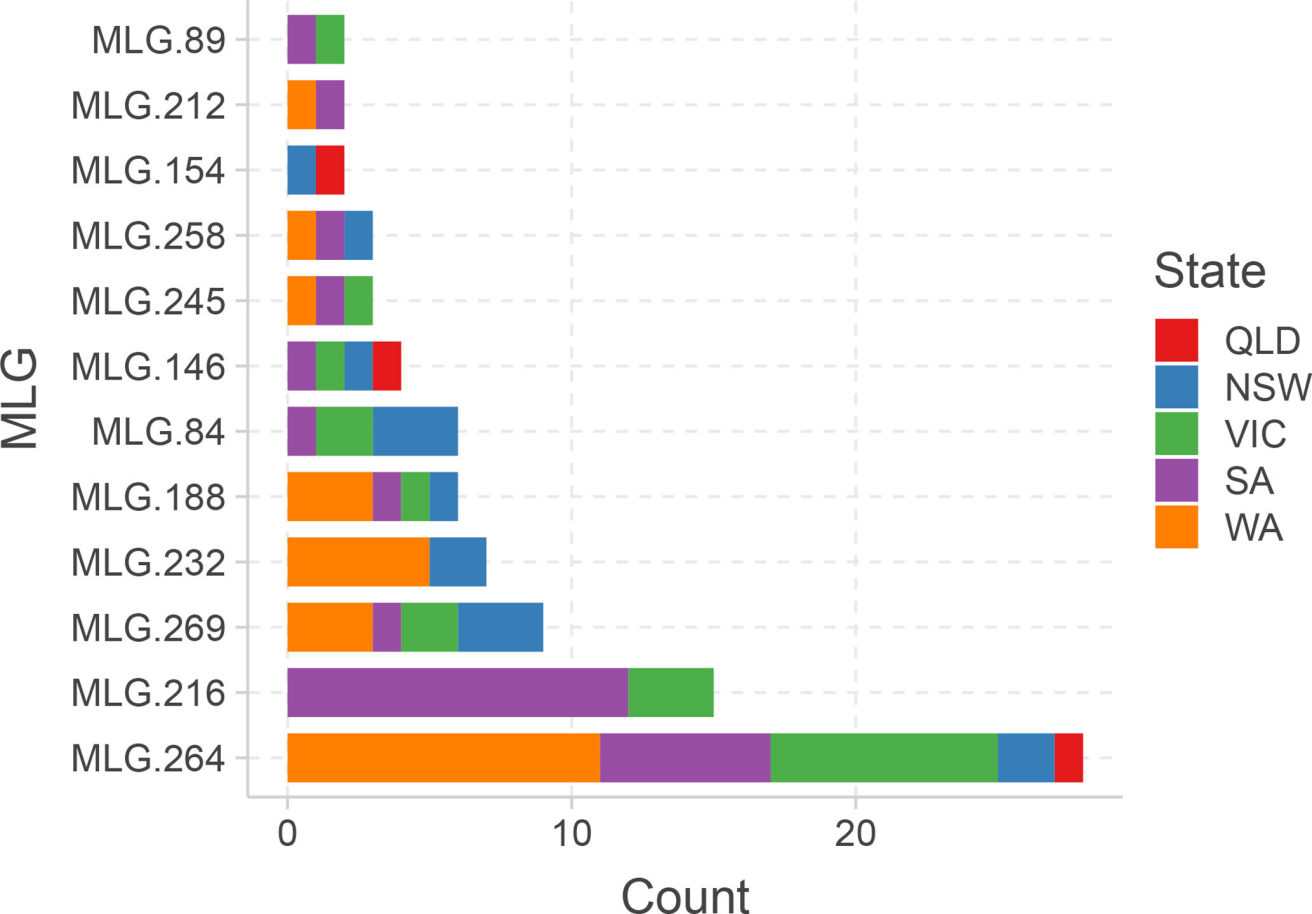
Multilocus genotypes of *A. rabiei* isolates sourced from multiple states in Australia, based on genome-wide SNP data.

A closer examination of several haplotypes highlighted differences in their occurrence, frequencies and distributions among states. For example, isolates of MLG.264 were abundant in WA (11 isolates, comprising 39.3 % of the isolates in the MLG) but these were also found in high frequencies in SA (eight isolates), and VIC (six isolates), and to a lesser extent in NSW (two isolates) and QLD (one isolate) (Fig. 3).

### Association between *A. rabiei* genotypes and pathogenicity

**Fig. 4.**
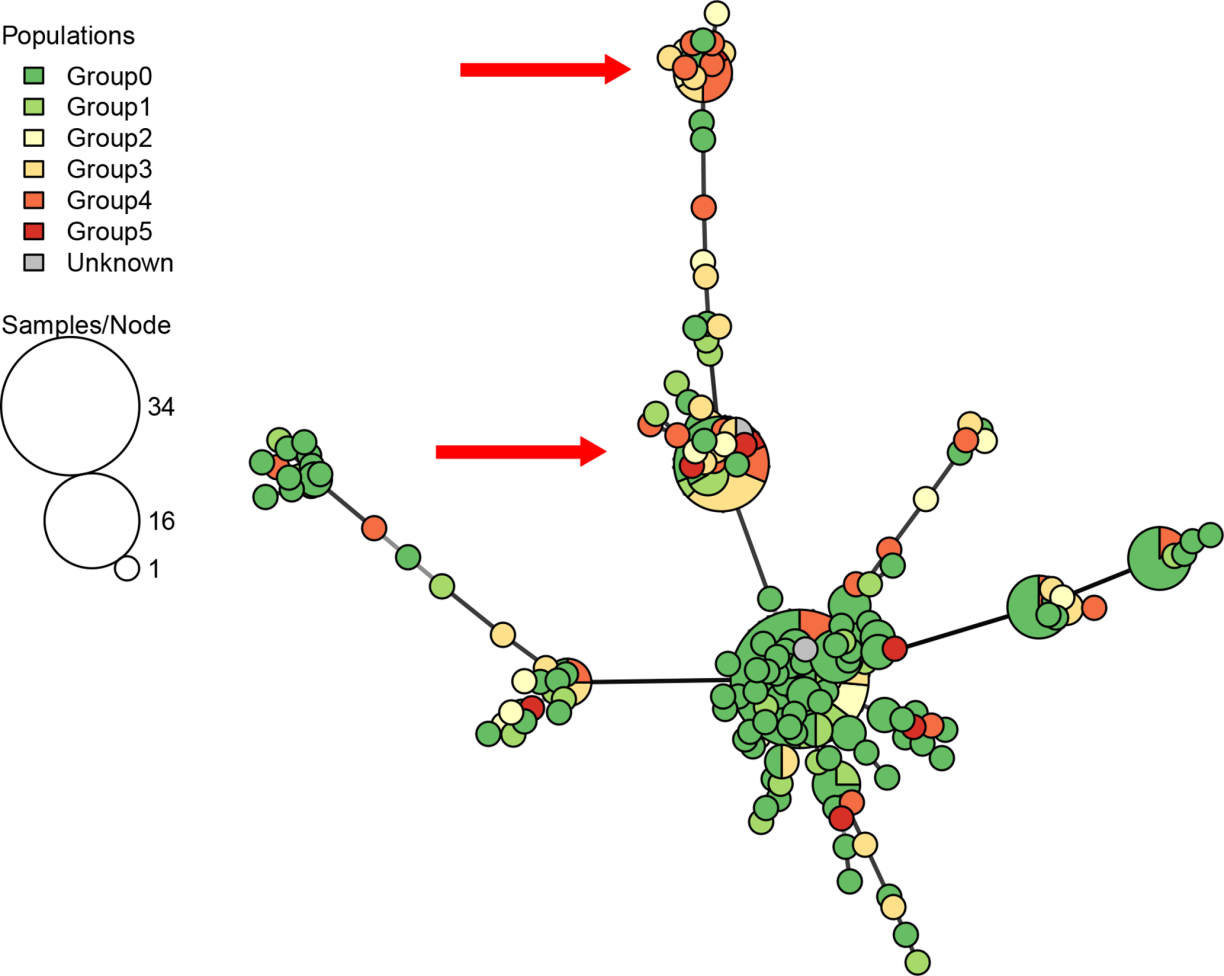
Minimum spanning network diagram of Australian *A. rabiei* multilocus genotypes by pathogenicity group. Where node size represents the number of isolates identified in each MLG, and the length of the branches between the nodes represents the genetic distance. Node colours represent the pathogenicity group of the isolates in the MLG. Arrows highlight nodes/clusters with high proportions of highly pathogenic isolates (PG ≥ 3).

The same MSN diagram of the MLG haplotypes, coloured to highlight the pathogenicity groups of the isolates, showed no distinct pattern of closely related highly pathogenic isolates, except for two diverged groups of isolates consisting mostly of isolates of pathogenicity groups 3–5 (red arrows in Fig. 4).

**Fig. 5.**
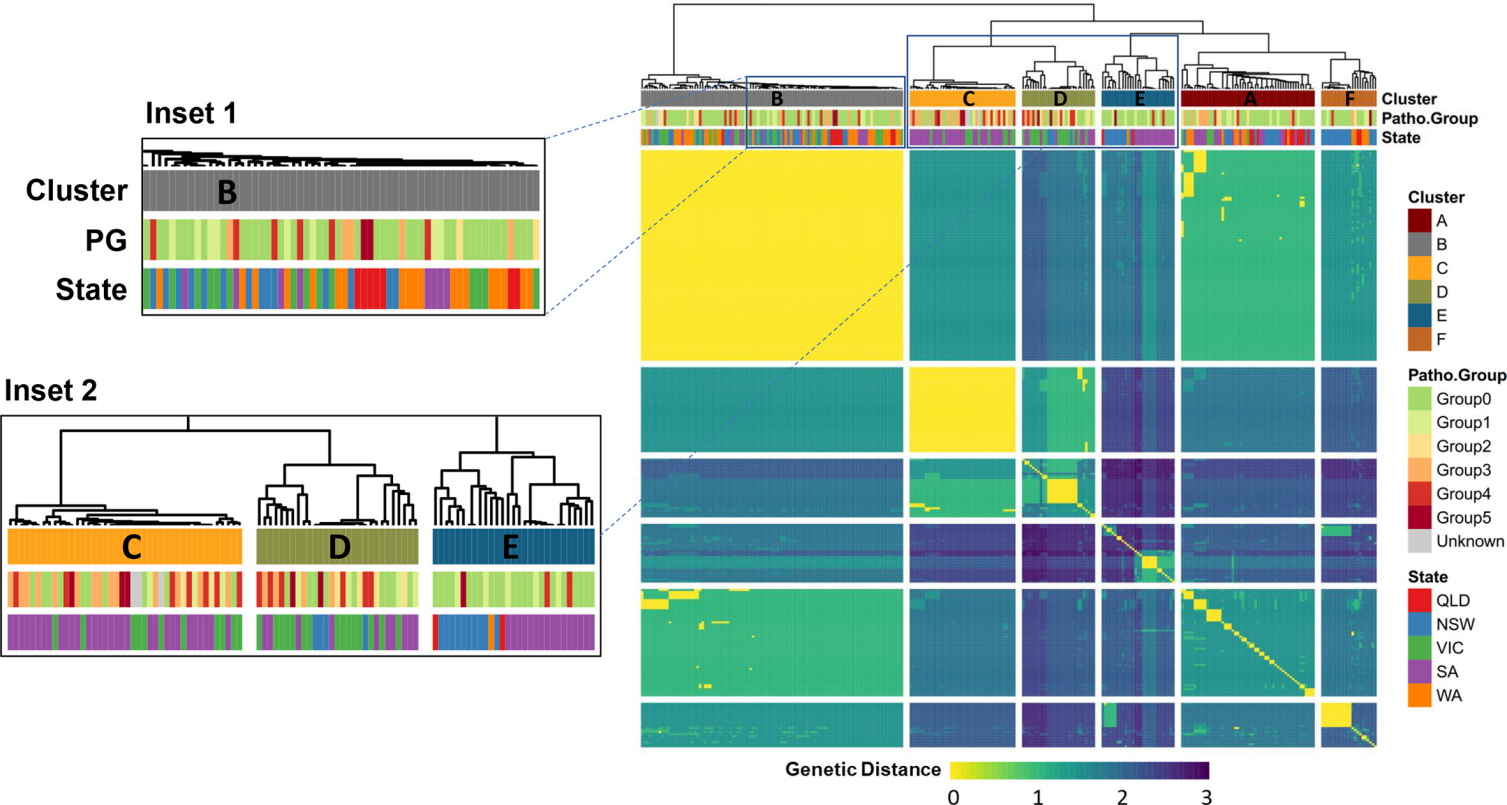
Genetic distance-based clustering of *A. rabiei* isolates. The heatmap and dendrogram are based on pairwise Euclidean distances that were calculated from SNP allele frequencies obtained from DArTseq data. Gaps in the heatmap separate between isolates clustered within major branches (denoted clusters A-F and coloured below the dendrogram according to the legend), as determined by hierarchical cluster analysis using the *hclust* function in R (default parameters used). Additional colour bars on top of the heatmap represent the pathogenicity group classification and state of origin of each isolate. Inset one shows a magnified view of genetically similar, but phenotypically diverse isolates in cluster B; Inset two shows a comparison between clusters C, D and E.

Visualizing the pairwise genetic distances between the Australian isolates as a clustered heatmap and dendrogram (Fig. 5) provided additional information to better interpret the level of relatedness between isolates and association with their state of origin and pathogenicity level.

Overall, the hierarchical clustering that was applied was in agreement with the MLG determination, with just 2 % of the MLGs including isolates from multiple clusters. MLGs 212, 232 and 245 included isolates from both clusters A and B, while MLG.174 comprised one isolate from cluster A and another from cluster F (Table S1). The large MLG (MLG.264) and its closely surrounding relatives, seen at the centre of Fig. 2 and in Fig. 3, are clearly identified as cluster B in the heatmap and dendrogram in Fig. 5. This cluster represents a range of isolates from several growing regions, years and pathogenicity groups (inset one in Fig. 5), although they were almost genetically identical (clonal) according to their MLG assignment.

In contrast, other clusters showed strong association with these descriptors. For example, cluster E (bottom inset in Fig. 5) comprised a distinct set of isolates originating from SA in 2013, along with NSW-originated isolates. Most of these cluster E isolates (~90 %) were of low pathogenicity (Table 4). Of further interest, cluster C comprised mostly high pathogenicity isolates, all from SA and VIC (inset two in Fig. 5). Based on the odds ratio, an isolate clustered within this group was at least ten times more likely to be classified as highly pathogenic (pathogenicity groups 3–5) than an isolate from clusters E or F, and at least six times more likely than an isolate from clusters A or B (Fig. 5, Table 4). A similar observation was made in the left branch of cluster D, although the isolates in this branch were less uniform genetically and the cluster included a branch of low-pathogenicity isolates (inset two in Fig. 5).

**Table 4.** Proportions of pathogenicity classification of *A. rabiei* isolates in dendrogram clusters

Cluster	High	Low*	Odds ratio†	Significance (*P*-value)
A	18.9 %	81.1 %	C/A=6.88	0.000877 (***)
B	19.4 %	80.6 %	C/B=6.64	6.73E-05 (***)
C	**61.5 %**	38.5 %	C/C=1	(ns)
D	41.4 %	58.6 %	C/D=2.26	0.575 (ns)
E	10.3 %	**89.7 %**	C/E=13.87	0.002061 (**)
F	13.6 %	86.4 %	C/F=10.13	0.012726 (*)

*Isolates were classified as ‘low’ or ‘high’ pathogenicity if they were classified in pathogenicity groups 0–2 or 3–5, respectively. Highest and lowest proportions are indicated in bold font.

†Differences in proportions of ‘low’ and ‘high’ pathogenicity isolates between clusters were determined by a generalized linear mixed-effect model and are presented as the odds ratio (OR) compared to cluster C, along with their statistical significance as validated by the Tukey method.

GWAS analysis, utilizing the same SNP set derived from the DArTseq data, revealed two SNP allelic variants, 44700443–64-A/G and 44702311–19 C/A, that were significantly associated with pathogenicity (qvalue≤0.05). These markers were annotated to their genomic coordinates and were located on contigs 06 and 20 of the *A. rabiei* ArME14 reference genome [32] (NCBI accessions RYYQ01000007.1 and RYYQ010000021.1, respectively, see Table 5).

**Table 5.** SNPs associated with pathogenicity levels in *A. rabiei*

SNP Name	Contig	Position	*P*-value	*q*-value
44700443–64-A/G	RYYQ01000021.1_ctg20	801 717	6.5431E^−10^	9.16034E^−09^
44702311–19 C/A	RYYQ01000007.1_ctg06	1 292 348	5.54659E^−05^	0.000388261

## Discussion

In this study, a genotyping-by-sequencing (GBS) approach was undertaken to assess the molecular genetic structure within the Australian *A. rabiei* population. Also, a select isolate core collection was interrogated to seek for potential correlation between isolate genotype and ability to cause significant disease severity on a differential host set.

### Population structure and diversity

This study and previous ones [37, 38] have verified the presence of a single mating-type (MAT1-2) and the absence of the second mating-type sequence (MAT 1–1), found elsewhere in the world, in the Australian *A. rabiei* population, indicating that the population is reproducing largely (if not solely) clonally. This mode of reproduction explains the low genetic diversity observed in Australia relative to other populations worldwide, as was demonstrated by analysis of simple sequence repeat (SSR) markers [8, 38, 39]. SSR markers are generally highly polymorphic, cost efficient to use and have been used extensively in population genetics studies [40]. High mutation rates and assumed neutral evolution of SSR loci result in the accumulation of numerous population-specific alleles, revealing hidden population structures. However, the high allelic variability of SSR markers may also mask patterns of genome-wide genetic diversity [41]. Furthermore, in a population with low genetic diversity, such as the Australian *A. rabiei* population, a larger number of SSR markers is required to accurately distinguish between individuals and to assign haplotypes. The development, validation and application of a large number of informative SSR markers is labour intensive, time consuming and costly [42].

As opposed to SSR markers, SNPs are widely spread across all regions of a genome, including within and in close proximity to gene coding regions and can be more readily applied to produce consistent heterozygosity and diversity estimates, such as Nei’s gene diversity (expected heterozygosity) than multi-allelic SSRs [41]. Genotyping using high-throughput sequencing technologies, such as whole-genome sequencing and GBS, allows an accurate identification of thousands of SNPs across entire or partial representations of genomes without prior knowledge of the genome (*de novo*). The relatively small size of the *A. rabiei* genome (~41 Mbp, GCA_004011695.1 [32]), means that these methods are affordable to use with large sample sizes for population studies and even high-resolution genome-wide association studies (GWAS), to associate variants with traits of interest [43].

The GBS approach applied in this study resulted with a fairly modest number of polymorphic markers, 212 SNPs in 185 MLGs, but still offered a high-resolution set of markers for the analysis of genetic relatedness among the Australian isolates, and enabled comparison to the ICARDA *A. rabiei* isolates. This provided a more accurate measure of the genetic relatedness within the Australian collection than the seven microsatellite markers previously used by Mehmood *et al.* [8]. The modest number of genome-wide polymorphic SNPs resulting from the DArTseq method in the current study, along with the level of clonality and genetic diversity metrics observed in the sampled population, demonstrated and confirmed the low level of genetic diversity previously reported for *A. rabiei* in Australia [8, 37, 38]. Minor seasonal and regional variations in genetic diversity were observed, which may be a result of the environmental conditions and scale of cropping affecting the presence of the pathogen in the growing regions and hence in the isolate collection.

Inclusion of the two isolates from the ICARDA collection provided context to the homogeneity of the genotype data from the Australian isolates and confirmed that the DArTseq method detected many more SNPs than would be evident from the assessment of the Australian isolate data alone. The low number of polymorphic SNP loci in the Australian *A. rabiei* population is a consequence of genetic homogeneity rather than any inefficiency of the DArTseq method for genotyping fungi for population studies.

The genetic distances between Australian isolates, within and between collection locations (Fig. 2 and inset two in Fig. 5) clearly showed that some isolates were highly related within a particular location, suggesting that they had evolved locally and potentially independently. In contrast, other MLGs represented isolates that may be considered as genetic clones, and these were sourced from multiple growing regions. This included MLG.146, MLG.188 and MLG.269, and MLG.264, which were sourced from all five states (Fig. 3). MLG.264 was identified in cluster B in Fig. 5 and was centrally positioned in Figs 2 and 3, connecting all other branches. MLG.264 comprised 28 isolates sourced from all growing regions, representing the ‘core’ clonal lineage of *A. rabiei* in Australia, from which other lines diverged. A comparison between the MLG determination presented in this study and the SSR-based haplotypes assigned by Mehmood *et al.* [9], revealed six MLGs that contained isolates assigned to multiple SSR haplotypes. One might hypothesize that MLG.264, which was tightly clustered in the current analysis, equates to the most frequently observed haplotype ARH01, observed from the SSR genotyping. However, the MLG.264 haplotype contained isolates previously assigned to five different SSR haplotypes. Furthermore, an examination of the isolates that were previously assigned to haplotype ARH01 revealed that they were mapped to 77 different DArTseq SNP-based MLGs, suggesting that the seven SSR loci previously used were indeed limited in their genotyping ability.

Conversely, 14 isolates that belonged to the same clonal lineage (MLG) were assigned to separate and distinct clusters. Among these, nine were collected in WA (64 %), at a frequency significantly higher than represented in the overall collection (17%). This is puzzling considering the high clonality of the population in WA, but it may provide evidence that WA isolates are derived from a few isolates that were imported from other regions and established within relatively few founder events during the expansion of the industry, thus explaining their genetic relatedness across clusters.

The presence of isolates from the same clonal lineages in multiple growing regions may be explained either by (1) limitation of the marker set resolution or (2) that the isolates were derived from the same clonal lineage and have been transported between regions. In total 185 MLGs were identified among the population isolates assessed, indicating the ability to differentiate close relatives. The latter hypothesis is further supported by the fact that no MLG clusters were found to comprise only isolates collected from WA or QLD, rather these were always found in association with isolates from other regions (Figs 2 and 5). Considering the geographic isolation of the chickpea growing region of WA, more than 1500 km from the nearest chickpea growing regions in SA, and the known natural dispersal distance of *A. rabiei* clonal spores (up to 100 m), it is highly unlikely that they have dispersed between the regions without human assistance [44, 45].

Furthermore, the higher level of genotype diversity observed among isolates collected in QLD (Table 3) and their spread into multiple distant MLGs, suggested that these isolates were ‘imported’ from other growing regions, probably through sourcing of contaminated seeds or spread by cropping equipment brought into the region. The chickpea industries in both QLD and WA have expanded rapidly in recent years, peaking in a ‘goldrush’ for chickpea production during the 2016 and 2017 growing seasons, with the grain prices reaching record highs and above US$700 per tonne. During the same period, seed was imported to QLD from various sources and a large proportion of seed was not certified as ‘clean seed’. In addition, crop rotation best practice was frequently not observed between consecutive seasons (KM, personal communications). The increase in cropping intensity and scale, along with lax cropping practices likely resulted in the additional selective pressure on the populations leading to the steep increase in frequency of highly pathogenic isolates, observed in QLD during the 2017 season [12].

### Pathogenicity of the Australian *A. rabiei* population

Despite the low genetic diversity observed and the reduced rate of chromosomal rearrangements expected by clonal propagation in the presence of a single mating type (MAT1-2), *A. rabiei* isolates in Australia exhibit a wide pathogenic range and are able to cause varying levels of disease on an established differential host set used to characterize them [8, 12]. These fit and adapted highly pathogenic isolates appear to be occurring at an ever-increasing rate, leading to shorter lifespans of newly bred and implemented resistant cultivars [6, 12]. According to McDonald and Linde [46], this adaptation process is likely to occur more rapidly in sexually reproducing pathogens in the presence of both mating types, resulting in increased pathogenic potential and epidemic risk [46]. Therefore, extreme biosecurity control measures should be taken to reduce the chance of introducing the second *A. rabiei* mating type (MAT1-1) into Australia.

The variant SNP markers that were identified as associated with pathogenicity group were annotated by their chromosomal coordinate and association with potential virulence genes, such as effector molecules, signalling and receptor molecules, and transcription factors. One of the pathogenicity-associated markers, 44700443–64-A/G on ArME14 contig 20, was co-located in a gene-rich region (NCBI Genebank accession https://www.ncbi.nlm.nih.gov/nuccore/KR139658.1), the solanapyrone biosynthesis gene cluster. This region includes genes related to metabolism, such as O-methyltransferase, dehydrogenase, polyketide synthase and transcription factors. Kim *et al*. [47] showed that knockdown of sol4, a novel type of Zn(II)2Cys6 zinc cluster transcription factor found in this region led to complete loss of solanapyrone biosynthesis, however this did not impact growth, sporulation or virulence of *A. rabiei* [47]. Further investigation of this gene cluster and the Tc1/Mariner-type transposable elements surrounding it may identify their role in the infection mechanism and possible link to important protein-altering variants. The second trait-associated genomic region indicated by marker 44 702 311 contained no genes that could be associated with pathogenicity.

Genotyping-by-sequencing (GBS) and particularly DArTseq has become a common practice for large-scale genotyping of agricultural crops, animals and other species, thanks to its affordability compared with whole genome sequencing and SNP-chip assays and its focus on ‘functional’ regions of the genome [16, 48–50]. GBS produces a set of markers throughout the genome, that is then used for population genetic analysis, genetic mapping, genome-wide association studies and other applications [51–53]. Despite its advantages, a known caveat of GBS is the high rate of missing data, caused by the random representation of loci in each sample, which limits the number of comparable markers present in all samples, leading to sample and locus dropout [54].

In the case of the highly clonal Australian *A. rabiei* population, DArTseq resulted in just 212 informative SNPs. This raises the question of whether the overall low population diversity and high clonality levels observed, particularly in clusters C and B, were a result of limited genotyping resolution or indicated a truly low level of genetic diversity. Previous studies have shown that a similar number of SNP markers spread over a small genome (41 Mbp for *A. rabiei* [32]) was sufficient to accurately determine population genetic structure, as was demonstrated for the fungal pathogen *Phytophthora rubi* [55].

To determine if the number of markers is sufficient to genetically distinguish between clonal lineages of *A. rabiei* and to obtain accurate marker-phenotype association to identify causative mutations in key genes, a higher marker density and coverage is required. The small genome size of *A. rabiei* and the continuous improvements in sequencing platforms along with reduction in sequencing prices are favourable to obtain this. In fact, a whole-genome sequencing or high-depth GBS approach to obtain high-density sets of markers from large sample sizes may well be within reach in coming years. This will enable genome-wide association studies to identify specific alleles and loci correlated with pathogenicity levels, which could assist in resistance chickpea breeding and the development of novel fungicides. A similar approach identified novel candidate genes for pathogenicity in the wheat fungal pathogen *Fusarium graminearum* using approximately 30 000 SNPs spread throughout its 36 Mbp genome [56].

The set of SNP markers used in this study identified clonal isolates that were separated by just a few allelic variants, but displayed a wide phenotypic diversity. This may have indicated that the marker resolution was not sufficient to identify markers directly associated with pathogenicity and suggested that an isolate’s pathogenicity was not necessarily determined by a SNP variant. Other mechanisms that may alter pathogenicity may include structural factors, such as variation in copy number of a virulence gene, rearrangement of genes in the genome, duplication and translocation of transposable elements, effector repertoire and gene expression modifications, as well as epigenetic effects caused by specific host–pathogen interactions [57, 58]. Identifying these variations may require other genomic approaches, such as pangenome comparisons incorporating long-read sequencing and transcriptomic approaches, including mRNA and short RNA sequencing [59, 60]. Application of these approaches in future studies will provide crucial evidence of genomic variations that are directly related to both pathogenicity and molecular interactions with the host resistance genes. The discovery of genetic cluster C, and to a lesser extent cluster D, that are indicative to a growing region and high pathogenicity, is encouraging in the search for distinct pathotype markers and provides a first step towards developing a molecular pathotype diagnostics method.

## Conclusions

This research aimed to elucidate the genetic structure of *A. rabiei* across the main chickpea growing regions of Australia and to determine any correlation of genotype with pathogenicity phenotype. To the best of our knowledge, this is the first published study that utilized a GBS approach and SNP markers to infer the genetic structure of an *A. rabiei* population, thus setting the foundation for ongoing investigation into specific genomic features of the pathogen that may assist Australian scientists, breeders, farmers and government agencies in managing and reducing the chickpea Ascochyta blight risk.

Although a direct genomic correlation to pathogenicity was not identified in the current study, an interesting pattern emerged suggesting that *A. rabiei* exhibits local micro-evolution within the broader growing region, likely driven by a founder effect, which has implications for local disease management and control of seed and farming equipment movements between the regions. The discovery of genetic clusters associated with highly pathogenic isolates holds a promise to the potential development of genetic markers that will enable early diagnostics of highly pathogenic *A. rabiei* isolates. This is crucial for accurate and informed disease management strategies.

These findings and further advances in the understanding of the pathogen populations will lead to the development of improved disease management strategies to allow sustainable production of chickpea as a source of food and income into the future.

## Supplementary Data

Supplementary material 1
