# Supplementary material for: Current population structure and pathogenicity patterns of Ascochyta rabiei in Australia
Source: Microb Genom. 2021 Jul 20;7(7):000627. doi: 10.1099/mgen.0.000627 (PMC8477395; doi:10.1099/mgen.0.000627)
Supplement: Supplementary material 1 [file mgen-7-0627-s001.pdf]

Supplementary Material

Isolate Data

Passport data for the isolates, including source (geographic and host cultivar), date of collection and host cultivar is provided in Table S1. The table also includes assigned Pathogenicity Group, SSR-based Haplotype, Multilocus Genotype (MLG), genetic Cluster and mating type (Mat.Type); see Methods section in the main manuscript for details of these descriptors.

Table S1. Details of *Ascochyta rabiei* isolates used in this study

Id	Location	State	Year	Host	Patho.Group	Haplotype	MLG	Cluster	Mat.Type
13MAR002	Marnoo	VIC	2013	PBA Slasher	Group0	Unknown	126	B	MAT1-2
13MUR002	Murtoa	VIC	2013	PBA Slasher	Group2	ARH01	249	A	MAT1-2
13RUP002	Rupanyup	VIC	2013	Genesis090	Group2	Unknown	254	C	MAT1-2
FT13092-1	Kingsford	SA	2013	Genesis090	Group4	ARH11	148	E	MAT1-2
FT13092-2	Kingsford	SA	2013	Genesis090	Group4	ARH04	71	E	MAT1-2
FT13092-3	Kingsford	SA	2013	Genesis090	Group1	Unknown	264	B	MAT1-2
FT13092-5	Kingsford	SA	2013	Genesis090	Group1	ARH01	146	B	MAT1-2
FT13093-1	Kingsford	SA	2013	Genesis090	Group1	ARH01	3	E	MAT1-2
FT13093-2	Kingsford	SA	2013	Genesis090	Group1	Unknown	35	E	MAT1-2
FT13093-3	Kingsford	SA	2013	Genesis090	Group1	Unknown	225	E	MAT1-2
FT13093-6	Kingsford	SA	2013	Genesis090	Group0	Unknown	134	A	MAT1-2
FT13095-1	Kingsford	SA	2013	CICA1152	Group0	ARH09	69	E	MAT1-2
FT13095-2	Kingsford	SA	2013	CICA1152	Group0	ARH01	65	E	MAT1-2
FT13095-3	Kingsford	SA	2013	CICA1152	Group0	Unknown	67	E	MAT1-2
FT13095-4	Kingsford	SA	2013	CICA1152	Group0	ARH01	226	E	MAT1-2
FT13095-5	Kingsford	SA	2013	CICA1152	Group0	ARH01	212	A	MAT1-2
FT13095-6	Kingsford	SA	2013	CICA1152	Group0	ARH01	221	E	MAT1-2
FT13096-1	Kingsford	SA	2013	CICA1152	Group0	Other	49	A	MAT1-2
FT13096-2	Kingsford	SA	2013	CICA1152	Group0	Unknown	103	A	MAT1-2
FT13096-3	Kingsford	SA	2013	CICA1152	Group0	Unknown	149	E	MAT1-2
FT13096-4	Kingsford	SA	2013	CICA1152	Group0	Unknown	68	E	MAT1-2
FT13096-5	Kingsford	SA	2013	CICA1152	Group0	ARH01	58	E	MAT1-2
FT13097-1	Kingsford	SA	2013	CICA1152	Group0	ARH01	66	E	MAT1-2
FT13097-2	Kingsford	SA	2013	CICA1152	Group0	ARH05	39	E	MAT1-2
FT13097-3	Kingsford	SA	2013	CICA1152	Group0	ARH03	105	A	MAT1-2
FT13097-4	Kingsford	SA	2013	CICA1152	Group0	ARH01	70	E	MAT1-2
14DON001	Donald	VIC	2014	PBA Slasher	Group0	ARH01	188	B	MAT1-2
14DON002	Donald	VIC	2014	PBA Slasher	Group0	ARH03	88	D	MAT1-2
14DON003	Donald	VIC	2014	PBA Slasher	Group1	ARH01	25	B	MAT1-2
14DON004	Donald	VIC	2014	PBA Slasher	Group1	Other	27	B	MAT1-2
14HOR014	Horsham	VIC	2014	Other	Group0	ARH01	76	A	MAT1-2
14HOR016	Horsham	VIC	2014	Other	Group0	ARH01	101	B	MAT1-2
14HOR017	Horsham	VIC	2014	Other	Group1	ARH01	264	B	MAT1-2
14HOR15	Horsham	VIC	2014	Other	Group0	ARH01	37	B	MAT1-2
F14038	Salter Springs	SA	2014	Monarch	Group0	ARH01	228	A	MAT1-2
F14039	Salter Springs	SA	2014	Monarch	Group0	ARH01	264	B	MAT1-2
F14040	Salter Springs	SA	2014	Monarch	Group0	ARH01	78	B	MAT1-2
F14090	Riverton	SA	2014	Other	Group0	ARH01	264	B	MAT1-2
TR6415	Yallaroi	NSW	2014	PBA HatTrick	Group4	ARH01	23	B	MAT1-2

Id	Location	State	Year	Host	Patho.Group	Haplotype	MLG	Cluster	Mat.Type
13MAR002	Marnoo	VIC	2013	PBA Slasher	Group0	Unknown	126	B	MAT1-2
13MUR002	Murtoa	VIC	2013	PBA Slasher	Group2	ARH01	249	A	MAT1-2
13RUP002	Rupanyup	VIC	2013	Genesis090	Group2	Unknown	254	C	MAT1-2
FT13092-1	Kingsford	SA	2013	Genesis090	Group4	ARH11	148	E	MAT1-2
FT13092-2	Kingsford	SA	2013	Genesis090	Group4	ARH04	71	E	MAT1-2
FT13092-3	Kingsford	SA	2013	Genesis090	Group1	Unknown	264	B	MAT1-2
FT13092-5	Kingsford	SA	2013	Genesis090	Group1	ARH01	146	B	MAT1-2
FT13093-1	Kingsford	SA	2013	Genesis090	Group1	ARH01	3	E	MAT1-2
FT13093-2	Kingsford	SA	2013	Genesis090	Group1	Unknown	35	E	MAT1-2
FT13093-3	Kingsford	SA	2013	Genesis090	Group1	Unknown	225	E	MAT1-2
FT13093-6	Kingsford	SA	2013	Genesis090	Group0	Unknown	134	A	MAT1-2
FT13095-1	Kingsford	SA	2013	CICA1152	Group0	ARH09	69	E	MAT1-2
TR6417	Yallaroi	NSW	2014	PBA HatTrick	Group4	ARH01	267	B	MAT1-2
TR6422	Yallaroi	NSW	2014	PBA HatTrick	Group1	ARH01	31	A	MAT1-2
TR6462	Gulargambone	NSW	2014	PBA HatTrick	Group0	Unknown	55	E	MAT1-2
TR6540	Tulloona	NSW	2014	PBA HatTrick	Group0	Unknown	45	B	MAT1-2
TR6545	Windridge	NSW	2014	PBA HatTrick	Group0	Unknown	115	F	MAT1-2
TR6546	Windridge	NSW	2014	PBA HatTrick	Group0	Unknown	29	E	MAT1-2
TR6601	Kindee	NSW	2014	PBA HatTrick	Group0	ARH01	62	E	MAT1-2
TR6602	Kindee	NSW	2014	PBA HatTrick	Group0	ARH01	63	E	MAT1-2
TR6620	Garah	NSW	2014	Other	Group0	ARH01	264	B	MAT1-2
TR6690	Gilgandra	NSW	2014	PBA HatTrick	Group0	Unknown	258	B	MAT1-2
TR6692	Gilgandra	NSW	2014	PBA HatTrick	Group0	Other	32	A	MAT1-2
TR6694	Gilgandra	NSW	2014	PBA HatTrick	Group0	Other	21	B	MAT1-2
TR6704	Trangie	NSW	2014	PBA HatTrick	Group0	ARH01	235	F	MAT1-2
TR6706	Narromine	NSW	2014	PBA HatTrick	Group0	Unknown	256	F	MAT1-2
TR6709	Narromine	NSW	2014	PBA HatTrick	Group0	Unknown	235	F	MAT1-2
TR6710	Narromine	NSW	2014	PBA HatTrick	Group0	Other	111	F	MAT1-2
TR6747	Peak Hill	NSW	2014	PBA HatTrick	Group0	Unknown	222	A	MAT1-2
TR6749	Peak Hill	NSW	2014	PBA HatTrick	Group0	ARH01	108	A	MAT1-2
TR6750	Peak Hill	NSW	2014	PBA HatTrick	Group0	ARH09	106	A	MAT1-2
TR6795	Gulargambone	NSW	2014	PBA HatTrick	Group0	Other	110	F	MAT1-2
TR6796	Gulargambone	NSW	2014	PBA HatTrick	Group0	Unknown	256	F	MAT1-2

Id	Location	State	Year	Host	Patho.Group	Haplotype	MLG	Cluster	Mat.Type
13MAR002	Marnoo	VIC	2013	PBA Slasher	Group0	Unknown	126	B	MAT1-2
13MUR002	Murtoa	VIC	2013	PBA Slasher	Group2	ARH01	249	A	MAT1-2
13RUP002	Rupanyup	VIC	2013	Genesis090	Group2	Unknown	254	C	MAT1-2
FT13092-1	Kingsford	SA	2013	Genesis090	Group4	ARH11	148	E	MAT1-2
FT13092-2	Kingsford	SA	2013	Genesis090	Group4	ARH04	71	E	MAT1-2
FT13092-3	Kingsford	SA	2013	Genesis090	Group1	Unknown	264	B	MAT1-2
FT13092-5	Kingsford	SA	2013	Genesis090	Group1	ARH01	146	B	MAT1-2
FT13093-1	Kingsford	SA	2013	Genesis090	Group1	ARH01	3	E	MAT1-2
FT13093-2	Kingsford	SA	2013	Genesis090	Group1	Unknown	35	E	MAT1-2
FT13093-3	Kingsford	SA	2013	Genesis090	Group1	Unknown	225	E	MAT1-2
FT13093-6	Kingsford	SA	2013	Genesis090	Group0	Unknown	134	A	MAT1-2
FT13095-1	Kingsford	SA	2013	CICA1152	Group0	ARH09	69	E	MAT1-2
TR6800	Armatree	NSW	2014	PBA HatTrick	Group0	Other	154	B	MAT1-2
TR6802	Armatree	NSW	2014	PBA HatTrick	Group0	ARH01	253	E	MAT1-2
15CUR002	Curyo	VIC	2015	Genesis090	Group4	Other	247	C	MAT1-2
15CUR003	Curyo	VIC	2015	Genesis090	Group1	ARH20	236	C	MAT1-2
15CUR004	Curyo	VIC	2015	Genesis090	Group1	ARH20	28	B	MAT1-2
15DON006	Donald	VIC	2015	PBA Slasher	Group1	ARH05	264	B	MAT1-2
15DON008	Donald	VIC	2015	PBA Slasher	Group4	ARH05	237	B	MAT1-2
15DON009	Donald	VIC	2015	PBA Slasher	Group4	ARH01	234	B	MAT1-2
15DON010	Donald	VIC	2015	PBA Slasher	Group4	ARH01	264	B	MAT1-2
F15009	Coonalpyn	SA	2015	PBA Striker	Group0	ARH01	152	D	MAT1-2
F15019	Crystal Brook	SA	2015	PBA Slasher	Group1	ARH01	152	D	MAT1-2
F15021	Crystal Brook	SA	2015	PBA Slasher	Group1	ARH01	95	D	MAT1-2
FT15001	Weetulta	SA	2015	Genesis090	Group1	ARH01	216	C	MAT1-2
FT15024	Moonta	SA	2015	Genesis090	Group4	ARH01	264	B	MAT1-2
FT15025	Moonta	SA	2015	Genesis090	Group2	ARH01	60	A	MAT1-2
FT15026	Moonta	SA	2015	Genesis090	Group4	ARH01	264	B	MAT1-2
FT15028	Weetulta	SA	2015	Genesis090	Group3	ARH04	96	C	MAT1-2
FT15029	Weetulta	SA	2015	Genesis090	Group3	ARH01	147	B	MAT1-2
16RUP002	Rupanyup	VIC	2016	Genesis090	Group0	ARH09	146	B	MAT1-2
16RUP012	Rupanyup	VIC	2016	Genesis090	Group1	ARH01	223	D	MAT1-2
16RUP013	Rupanyup	VIC	2016	Genesis090	Group1	ARH01	9	B	MAT1-2
16RUP014	Rupanyup	VIC	2016	Genesis090	Group2	ARH01	85	D	MAT1-2
ARD-16-650	Ardath	WA	2016	PBA Striker	Group0	Unknown	42	A	MAT1-2
ARD-16-651	Ardath	WA	2016	PBA Striker	Group0	ARH01	22	B	MAT1-2
ARD-16-655	Ardath	WA	2016	PBA Striker	Group0	ARH01	264	B	MAT1-2
ARD-16-657	Ardath	WA	2016	PBA Striker	Group0	Unknown	56	B	MAT1-2
ARD-16-658	Ardath	WA	2016	PBA Striker	Group0	ARH01	229	B	MAT1-2
DON-16-629	Dongara	WA	2016	Other	Group0	Unknown	232	A	MAT1-2
DON-16-638	Dongara	WA	2016	Other	Group0	ARH11	179	B	MAT1-2
DON-16-643	Dongara	WA	2016	Other	Group0	ARH23	177	B	MAT1-2
F16083-1	Moonta	SA	2016	Genesis090	Group3	ARH01	54	C	MAT1-2
F16084-1	Kadina	SA	2016	Genesis090	Group4	ARH01	216	C	MAT1-2
F16149-1	Condowie	SA	2016	Genesis090	Group4	ARH01	72	C	MAT1-2
F16152-1	Nuriootpa	SA	2016	Genesis090	Group0	Other	216	C	MAT1-2
F16156-1	Berriwillock	SA	2016	Genesis090	Group0	ARH01	216	C	MAT1-2
F16165-1	Grace Plains	SA	2016	Other	Group4	ARH01	57	C	MAT1-2
F16181-1	Melton	SA	2016	Genesis090	Group0	Unknown	74	C	MAT1-2
F16186-1	Boort	VIC	2016	Genesis090	Unknown	Unknown	216	C	MAT1-2
F16188-1	Melton	SA	2016	Other	Group0	Unknown	90	D	MAT1-2

Id	Location	State	Year	Host	Patho.Group	Haplotype	MLG	Cluster	Mat.Type
13MAR002	Marnoo	VIC	2013	PBA Slasher	Group0	Unknown	126	B	MAT1-2
13MUR002	Murtoa	VIC	2013	PBA Slasher	Group2	ARH01	249	A	MAT1-2
13RUP002	Rupanyup	VIC	2013	Genesis090	Group2	Unknown	254	C	MAT1-2
FT13092-1	Kingsford	SA	2013	Genesis090	Group4	ARH11	148	E	MAT1-2
FT13092-2	Kingsford	SA	2013	Genesis090	Group4	ARH04	71	E	MAT1-2
FT13092-3	Kingsford	SA	2013	Genesis090	Group1	Unknown	264	B	MAT1-2
FT13092-5	Kingsford	SA	2013	Genesis090	Group1	ARH01	146	B	MAT1-2
FT13093-1	Kingsford	SA	2013	Genesis090	Group1	ARH01	3	E	MAT1-2
FT13093-2	Kingsford	SA	2013	Genesis090	Group1	Unknown	35	E	MAT1-2
FT13093-3	Kingsford	SA	2013	Genesis090	Group1	Unknown	225	E	MAT1-2
FT13093-6	Kingsford	SA	2013	Genesis090	Group0	Unknown	134	A	MAT1-2
FT13095-1	Kingsford	SA	2013	CICA1152	Group0	ARH09	69	E	MAT1-2
F16207-1	Pt Broughton	SA	2016	Genesis090	Group4	ARH01	97	A	MAT1-2
F16253-1	Pt Broughton	SA	2016	Genesis090	Group3	ARH01	61	C	MAT1-2
F16256-1	Daveyston	SA	2016	Genesis090	Group0	ARH01	19	B	MAT1-2
F16313-1	Coonalpyn	SA	2016	Genesis090	Group3	ARH01	94	C	MAT1-2
MGW-16-644	Mingenew	WA	2016	Other	Group0	Unknown	176	A	MAT1-2
MGW-16-645	Mingenew	WA	2016	Other	Group0	Unknown	178	A	MAT1-2
MGW-16-646	Mingenew	WA	2016	Other	Group0	ARH01	264	B	MAT1-2
MGW-16-647	Mingenew	WA	2016	Other	Group0	Unknown	188	B	MAT1-2
MGW-16-648	Mingenew	WA	2016	Other	Group0	ARH23	232	B	MAT1-2
TR8105	Narromine	NSW	2016	PBA HatTrick	Group1	ARH01	153	E	MAT1-2
TR8334	Brookstead	QLD	2016	PBA HatTrick	Group0	ARH01	107	A	MAT1-2
TR8336	Brookstead	QLD	2016	PBA HatTrick	Group0	ARH01	100	E	MAT1-2
TR8383	Munwonga	NSW	2016	PBA HatTrick	Group1	Unknown	128	A	MAT1-2
TR8384	Munwonga	NSW	2016	PBA HatTrick	Group1	ARH01	121	A	MAT1-2
TR8387	Munwonga	NSW	2016	PBA HatTrick	Group0	ARH01	1	A	MAT1-2
TR8392	Garah	NSW	2016	PBA HatTrick	Group0	Unknown	11	F	MAT1-2
TR8653	Merwood, Croppa Ck	QLD	2016	PBA Seamer	Group0	ARH01	146	B	MAT1-2
TR8655	Merwood, Croppa Ck	QLD	2016	PBA Seamer	Group0	ARH01	6	B	MAT1-2
TR8657	Merwood, Croppa Ck	QLD	2016	PBA Seamer	Group0	ARH09	264	B	MAT1-2
TR8744	Dalby	NSW	2016	PBA Seamer	Group1	ARH09	145	E	MAT1-2
TR8749	Dalby	NSW	2016	PBA Seamer	Group0	Unknown	188	B	MAT1-2
TR8750	Dalby	NSW	2016	PBA Seamer	Group0	ARH01	77	F	MAT1-2
122/17-1	Wasleys	SA	2017	Genesis090	Group0	Unknown	246	A	MAT1-2
122/17-2	Wasleys	SA	2017	Genesis090	Group0	ARH01	264	B	MAT1-2

Id	Location	State	Year	Host	Patho.Group	Haplotype	MLG	Cluster	Mat.Type
13MAR002	Marnoo	VIC	2013	PBA Slasher	Group0	Unknown	126	B	MAT1-2
13MUR002	Murtoa	VIC	2013	PBA Slasher	Group2	ARH01	249	A	MAT1-2
13RUP002	Rupanyup	VIC	2013	Genesis090	Group2	Unknown	254	C	MAT1-2
FT13092-1	Kingsford	SA	2013	Genesis090	Group4	ARH11	148	E	MAT1-2
FT13092-2	Kingsford	SA	2013	Genesis090	Group4	ARH04	71	E	MAT1-2
FT13092-3	Kingsford	SA	2013	Genesis090	Group1	Unknown	264	B	MAT1-2
FT13092-5	Kingsford	SA	2013	Genesis090	Group1	ARH01	146	B	MAT1-2
FT13093-1	Kingsford	SA	2013	Genesis090	Group1	ARH01	3	E	MAT1-2
FT13093-2	Kingsford	SA	2013	Genesis090	Group1	Unknown	35	E	MAT1-2
FT13093-3	Kingsford	SA	2013	Genesis090	Group1	Unknown	225	E	MAT1-2
FT13093-6	Kingsford	SA	2013	Genesis090	Group0	Unknown	134	A	MAT1-2
FT13095-1	Kingsford	SA	2013	CICA1152	Group0	ARH09	69	E	MAT1-2
122/17-3	Wasleys	SA	2017	Genesis090	Group0	ARH01	258	B	MAT1-2
17CUR001	Curyo	VIC	2017	Genesis090	Group0	ARH01	245	B	MAT1-2
17CUR002	Curyo	VIC	2017	Genesis090	Group0	ARH09	197	D	MAT1-2
17CUR005	Curyo	VIC	2017	Genesis090	Group5	ARH01	10	D	MAT1-2
17CUR007	Curyo	VIC	2017	Genesis090	Group3	ARH01	255	D	MAT1-2
17CUR009	Curyo	VIC	2017	Genesis090	Group1	ARH01	205	A	MAT1-2
17CUR013	Curyo	VIC	2017	Genesis090	Group0	ARH09	216	C	MAT1-2
17HOR001	Horsham	VIC	2017	Genesis090	Group0	Unknown	269	B	MAT1-2
17HOR009	Horsham	VIC	2017	Genesis090	Group0	Unknown	224	B	MAT1-2
A17061	Curyo, VIC	VIC	2017	Other	Group0	ARH01	248	C	MAT1-2
A17062	Curyo, VIC	VIC	2017	Other	Group0	ARH01	73	D	MAT1-2
A17066	Curyo, VIC	VIC	2017	Other	Group2	Other	264	B	MAT1-2
A17069	Curyo, VIC	VIC	2017	Other	Group0	ARH01	41	B	MAT1-2
F17067-1	Coonalpyn	SA	2017	Genesis090	Group0	ARH01	104	B	MAT1-2
F17076-1	Finley	NSW	2017	Genesis090	Group4	ARH01	84	D	MAT1-2
F17077-1	Bute	SA	2017	Genesis090	Group2	Unknown	34	D	MAT1-2
F17081-1	Wasleys	SA	2017	Genesis090	Group1	ARH01	75	C	MAT1-2
F17165-1	Blyth	SA	2017	Genesis090	Group3	ARH01	84	D	MAT1-2
F17175-1	Elmore	VIC	2017	Genesis090	Group0	ARH01	40	C	MAT1-2
F17175-2	Elmore	VIC	2017	Genesis090	Unknown	Unknown	216	C	MAT1-2
F17175-3	Elmore	VIC	2017	Genesis090	Unknown	Unknown	89	C	MAT1-2
F17191-1	Pt Broughton	SA	2017	Genesis090	Group5	ARH01	227	C	MAT1-2
F17191-2	Pt Broughton	SA	2017	Genesis090	Group5	ARH01	216	C	MAT1-2
F17191-3	Pt Broughton	SA	2017	Genesis090	Group5	Unknown	16	C	MAT1-2
F17200-1	Balaklava	SA	2017	Other	Group0	ARH01	216	C	MAT1-2
F17200-2	Balaklava	SA	2017	Other	Group3	ARH01	216	C	MAT1-2
F17200-3	Balaklava	SA	2017	Other	Group3	ARH01	89	C	MAT1-2
F17201-1	Balaklava	SA	2017	Other	Group0	ARH01	120	A	MAT1-2
MER-17-373	Merredin	WA	2017	PBA Striker	Group0	Unknown	264	B	MAT1-2
MER-17-374	Merredin	WA	2017	PBA Striker	Group1	ARH01	245	B	MAT1-2
MER-17-376	Merredin	WA	2017	PBA Striker	Group1	ARH03	264	B	MAT1-2
MER-17-378	Merredin	WA	2017	PBA Striker	Group3	ARH03	232	A	MAT1-2
MER-17-379	Merredin	WA	2017	PBA Striker	Group0	Unknown	102	B	MAT1-2
MER-17-380	Merredin	WA	2017	PBA Striker	Group2	ARH01	164	B	MAT1-2
MER-17-381	Merredin	WA	2017	PBA Striker	Group0	Unknown	18	B	MAT1-2
MER-17-382	Merredin	WA	2017	PBA Striker	Group0	Unknown	264	B	MAT1-2
MIL17253	Mullewa	WA	2017	Other	Group3	Unknown	264	B	MAT1-2
MIL17257	Mullewa	WA	2017	Other	Group0	Unknown	4	B	MAT1-2
MIL17259	Mullewa	WA	2017	Other	Group3	Unknown	174	F	MAT1-2
TR9529	Chinchilla	QLD	2017	PBA Seamer	Group5	Unknown	51	B	MAT1-2

Id	Location	State	Year	Host	Patho.Group	Haplotype	MLG	Cluster	Mat.Type
13MAR002	Marnoo	VIC	2013	PBA Slasher	Group0	Unknown	126	B	MAT1-2
13MUR002	Murtoa	VIC	2013	PBA Slasher	Group2	ARH01	249	A	MAT1-2
13RUP002	Rupanyup	VIC	2013	Genesis090	Group2	Unknown	254	C	MAT1-2
FT13092-1	Kingsford	SA	2013	Genesis090	Group4	ARH11	148	E	MAT1-2
FT13092-2	Kingsford	SA	2013	Genesis090	Group4	ARH04	71	E	MAT1-2
FT13092-3	Kingsford	SA	2013	Genesis090	Group1	Unknown	264	B	MAT1-2
FT13092-5	Kingsford	SA	2013	Genesis090	Group1	ARH01	146	B	MAT1-2
FT13093-1	Kingsford	SA	2013	Genesis090	Group1	ARH01	3	E	MAT1-2
FT13093-2	Kingsford	SA	2013	Genesis090	Group1	Unknown	35	E	MAT1-2
FT13093-3	Kingsford	SA	2013	Genesis090	Group1	Unknown	225	E	MAT1-2
FT13093-6	Kingsford	SA	2013	Genesis090	Group0	Unknown	134	A	MAT1-2
FT13095-1	Kingsford	SA	2013	CICA1152	Group0	ARH09	69	E	MAT1-2
TR9530	Dulacca, Chinchilla	QLD	2017	PBA Seamer	Group0	Unknown	53	B	MAT1-2
TR9531	Dulacca, Chinchilla	QLD	2017	PBA Seamer	Group0	ARH01	50	F	MAT1-2
TR9532	Chinchilla	QLD	2017	PBA Seamer	Group3	Unknown	154	B	MAT1-2
TR9533	Chinchilla	QLD	2017	PBA Seamer	Group4	Unknown	124	A	MAT1-2
TR9534	Dalby	QLD	2017	PBA Seamer	Group0	Unknown	268	A	MAT1-2
TR9535	Gravel Pit Hill	QLD	2017	PBA Seamer	Group0	Unknown	109	A	MAT1-2
TR9536	Gravel Pit Hill	QLD	2017	PBA Seamer	Group5	Unknown	17	B	MAT1-2
TR9537	Gravel Pit Hill	QLD	2017	PBA Seamer	Group0	ARH01	138	A	MAT1-2
TR9538	Gravel Pit Hill	QLD	2017	PBA Seamer	Group0	Unknown	30	E	MAT1-2
TR9539	Fox Holes	QLD	2017	PBA Seamer	Group0	Unknown	251	F	MAT1-2
TR9540	Fox Holes	QLD	2017	PBA Seamer	Group0	Unknown	12	A	MAT1-2
TR9541	Fox Holes	QLD	2017	PBA Seamer	Group0	Unknown	231	B	MAT1-2
TR9542	Fox Holes	QLD	2017	PBA Seamer	Group0	Unknown	250	A	MAT1-2
TR9543	Fox Holes	QLD	2017	PBA Seamer	Group3	Unknown	59	A	MAT1-2
TR9544	Fox Holes	QLD	2017	PBA Seamer	Group0	Unknown	48	A	MAT1-2
TR9568	Gurley	NSW	2017	PBA Seamer	Group3	ARH09	117	B	MAT1-2
TR9571	Gurley	NSW	2017	PBA Seamer	Group5	ARH09	5	E	MAT1-2
TR9572	Gurley	NSW	2017	PBA Seamer	Group4	ARH01	235	F	MAT1-2
TR9573	Gurley	NSW	2017	PBA Seamer	Group5	ARH01	64	F	MAT1-2
TR9700	Walgett	NSW	2017	PBA HatTrick	Group0	ARH05	7	A	MAT1-2
TR9701	Walgett	NSW	2017	PBA HatTrick	Group0	ARH01	110	F	MAT1-2

Id	Location	State	Year	Host	Patho.Group	Haplotype	MLG	Cluster	Mat.Type
13MAR002	Marnoo	VIC	2013	PBA Slasher	Group0	Unknown	126	B	MAT1-2
13MUR002	Murtoa	VIC	2013	PBA Slasher	Group2	ARH01	249	A	MAT1-2
13RUP002	Rupanyup	VIC	2013	Genesis090	Group2	Unknown	254	C	MAT1-2
FT13092-1	Kingsford	SA	2013	Genesis090	Group4	ARH11	148	E	MAT1-2
FT13092-2	Kingsford	SA	2013	Genesis090	Group4	ARH04	71	E	MAT1-2
FT13092-3	Kingsford	SA	2013	Genesis090	Group1	Unknown	264	B	MAT1-2
FT13092-5	Kingsford	SA	2013	Genesis090	Group1	ARH01	146	B	MAT1-2
FT13093-1	Kingsford	SA	2013	Genesis090	Group1	ARH01	3	E	MAT1-2
FT13093-2	Kingsford	SA	2013	Genesis090	Group1	Unknown	35	E	MAT1-2
FT13093-3	Kingsford	SA	2013	Genesis090	Group1	Unknown	225	E	MAT1-2
FT13093-6	Kingsford	SA	2013	Genesis090	Group0	Unknown	134	A	MAT1-2
FT13095-1	Kingsford	SA	2013	CICA1152	Group0	ARH09	69	E	MAT1-2
TR9702	Walgett	NSW	2017	PBA Seamer	Group0	ARH01	235	F	MAT1-2
TR9703	Walgett	NSW	2017	PBA Seamer	Group0	ARH01	8	B	MAT1-2
TR9704	Walgett	NSW	2017	PBA Seamer	Unknown	ARH01	139	B	MAT1-2
TR9706	Walgett	NSW	2017	PBA Seamer	Group0	ARH01	14	B	MAT1-2
TR9707	Walgett	NSW	2017	PBA Seamer	Group0	ARH01	36	D	MAT1-2
TR9708	Walgett	NSW	2017	PBA Seamer	Group5	ARH01	84	D	MAT1-2
TR9709	Walgett	NSW	2017	PBA Seamer	Group0	ARH03	84	D	MAT1-2
TR9710	Walgett	NSW	2017	PBA Seamer	Group4	ARH01	264	B	MAT1-2
TR9712	Walgett	NSW	2017	PBA Seamer	Group2	ARH01	52	B	MAT1-2
TR9713	Walgett	NSW	2017	PBA Seamer	Group3	ARH01	232	A	MAT1-2
158-1/18	Freeling	SA	2018	Genesis090	Group3	Unknown	38	C	MAT1-2
158-2/18	Freeling	SA	2018	Genesis090	Group4	Unknown	87	C	MAT1-2
18BRK1-1-01	Bruce Rock	WA	2018	PBA Striker	Group1	Unknown	264	B	MAT1-2
18BRK1-1-02	Bruce Rock	WA	2018	PBA Striker	Group0	Unknown	188	B	MAT1-2
18BRK1-1-03	Bruce Rock	WA	2018	PBA Striker	Group0	Unknown	232	A	MAT1-2
18BRK1-3-01	Bruce Rock	WA	2018	PBA Striker	Group3	Unknown	269	B	MAT1-2
18BRK1-3-02	Bruce Rock	WA	2018	PBA Striker	Group0	Unknown	22	B	MAT1-2
18BRK1-3-03	Bruce Rock	WA	2018	PBA Striker	Group0	Unknown	264	B	MAT1-2
18BRK2-1-01	Bruce Rock	WA	2018	PBA Slasher	Group0	Unknown	119	E	MAT1-2
18MGW1-4-01	Mingenew NVT	WA	2018	Maiden	Group2	Unknown	264	B	MAT1-2
18MGW1-5-01	Mingenew NVT	WA	2018	Maiden	Group0	Unknown	188	B	MAT1-2
18MGW1-6-01	Mingenew NVT	WA	2018	Maiden	Group0	Unknown	196	A	MAT1-2
18MGW1-8-01	Mingenew NVT	WA	2018	PBA Slasher	Group0	Unknown	212	B	MAT1-2
18MGW1-8-02	Mingenew NVT	WA	2018	PBA Slasher	Group0	Unknown	269	B	MAT1-2
18MGW1-9-01	Mingenew NVT	WA	2018	Other	Group0	Unknown	269	B	MAT1-2

Id	Location	State	Year	Host	Patho.Group	Haplotype	MLG	Cluster	Mat.Type
13MAR002	Marnoo	VIC	2013	PBA Slasher	Group0	Unknown	126	B	MAT1-2
13MUR002	Murtoa	VIC	2013	PBA Slasher	Group2	ARH01	249	A	MAT1-2
13RUP002	Rupanyup	VIC	2013	Genesis090	Group2	Unknown	254	C	MAT1-2
FT13092-1	Kingsford	SA	2013	Genesis090	Group4	ARH11	148	E	MAT1-2
FT13092-2	Kingsford	SA	2013	Genesis090	Group4	ARH04	71	E	MAT1-2
FT13092-3	Kingsford	SA	2013	Genesis090	Group1	Unknown	264	B	MAT1-2
FT13092-5	Kingsford	SA	2013	Genesis090	Group1	ARH01	146	B	MAT1-2
FT13093-1	Kingsford	SA	2013	Genesis090	Group1	ARH01	3	E	MAT1-2
FT13093-2	Kingsford	SA	2013	Genesis090	Group1	Unknown	35	E	MAT1-2
FT13093-3	Kingsford	SA	2013	Genesis090	Group1	Unknown	225	E	MAT1-2
FT13093-6	Kingsford	SA	2013	Genesis090	Group0	Unknown	134	A	MAT1-2
FT13095-1	Kingsford	SA	2013	CICA1152	Group0	ARH09	69	E	MAT1-2
18MLW1-2-01	Mullewa NVT	WA	2018	Maiden	Group0	Unknown	232	B	MAT1-2
18MLW1-2-02	Mullewa NVT	WA	2018	Maiden	Group2	Unknown	174	A	MAT1-2
18MLW1-2-03	Mullewa NVT	WA	2018	Maiden	Group4	Unknown	209	A	MAT1-2
18NBN2-1-01	Narembeen	WA	2018	PBA Slasher	Group0	Unknown	194	B	MAT1-2
18NBN2-1-02	Narembeen	WA	2018	PBA Slasher	Group0	Unknown	264	B	MAT1-2
18NBN2-1-03	Narembeen	WA	2018	PBA Slasher	Group0	Unknown	258	B	MAT1-2
18NBN2-1-05	Narembeen	WA	2018	PBA Slasher	Group0	Unknown	264	B	MAT1-2
18TSP1-1-01	Three Springs NVT	WA	2018	Other	Group1	Unknown	185	F	MAT1-2
18TSP1-1-02	Three Springs NVT	WA	2018	Other	Group1	Unknown	186	F	MAT1-2
18TSP1-3-01	Three Springs NVT	WA	2018	Other	Group0	Unknown	185	F	MAT1-2
18TSP1-4-02	Three Springs NVT	WA	2018	Other	Group2	Unknown	184	F	MAT1-2
83-1/18	Kulpara	SA	2018	Genesis090	Group2	Unknown	98	A	MAT1-2
83-2/18	Kulpara	SA	2018	Genesis090	Group2	Unknown	99	A	MAT1-2
AS17083	WRS Paddock 4	VIC	2018	PBA Striker	Group4	Unknown	84	D	MAT1-2
AS17087	Curyo SPA	VIC	2018	Genesis090	Group4	Unknown	244	D	MAT1-2
AS18079	WRS Bay H	VIC	2018	PBA Striker	Group4	Unknown	261	C	MAT1-2
AS18080	WRS Bay H	VIC	2018	Genesis090	Group0	Unknown	169	A	MAT1-2
AS18082	WRS Paddock 4	VIC	2018	Genesis090	Group2	Unknown	264	B	MAT1-2
AS18084	WRS Paddock 5	VIC	2018	Monarch	Group2	Unknown	265	B	MAT1-2
AS18086	Curyo SPA	VIC	2018	PBA Striker	Group1	Unknown	195	D	MAT1-2
AS18092	Birchip NVT	VIC	2018	Monarch	Group4	Unknown	189	D	MAT1-2
AS18098	Birchip NVT	VIC	2018	PBA Striker	Group4	Unknown	264	B	MAT1-2
AS18105	Taranyurk NVT	VIC	2018	PBA Striker	Group0	Unknown	264	B	MAT1-2
AS18115	Telangatuk SPA	VIC	2018	Genesis090	Group0	Unknown	243	B	MAT1-2
AS18116	Kaniva NVT	VIC	2018	Monarch	Group3	Unknown	264	B	MAT1-2
AS18128	Horsham SPA	VIC	2018	Genesis090	Group3	Unknown	170	A	MAT1-2
AS18163	Wimmera Survey Paddock 20	VIC	2018	Genesis090	Group0	Unknown	181	D	MAT1-2

Id	Location	State	Year	Host	Patho.Group	Haplotype	MLG	Cluster	Mat.Type
13MAR002	Marnoo	VIC	2013	PBA Slasher	Group0	Unknown	126	B	MAT1-2
13MUR002	Murtoa	VIC	2013	PBA Slasher	Group2	ARH01	249	A	MAT1-2
13RUP002	Rupanyup	VIC	2013	Genesis090	Group2	Unknown	254	C	MAT1-2
FT13092-1	Kingsford	SA	2013	Genesis090	Group4	ARH11	148	E	MAT1-2
FT13092-2	Kingsford	SA	2013	Genesis090	Group4	ARH04	71	E	MAT1-2
FT13092-3	Kingsford	SA	2013	Genesis090	Group1	Unknown	264	B	MAT1-2
FT13092-5	Kingsford	SA	2013	Genesis090	Group1	ARH01	146	B	MAT1-2
FT13093-1	Kingsford	SA	2013	Genesis090	Group1	ARH01	3	E	MAT1-2
FT13093-2	Kingsford	SA	2013	Genesis090	Group1	Unknown	35	E	MAT1-2
FT13093-3	Kingsford	SA	2013	Genesis090	Group1	Unknown	225	E	MAT1-2
FT13093-6	Kingsford	SA	2013	Genesis090	Group0	Unknown	134	A	MAT1-2
FT13095-1	Kingsford	SA	2013	CICA1152	Group0	ARH09	69	E	MAT1-2
AS18165	Wimmera Survey Paddock 8	VIC	2018	Genesis090	Group3	Unknown	190	C	MAT1-2
AS18166	Mallee Survey Paddock 17	VIC	2018	Genesis090	Group3	Unknown	269	B	MAT1-2
AS18167	Mallee Survey Paddock 14	VIC	2018	Genesis090	Group4	Unknown	259	D	MAT1-2
AS18168	Mallee Survey Paddock 10	VIC	2018	Genesis090	Group1	Unknown	26	B	MAT1-2
AS18169	Mallee Survey Paddock 9	VIC	2018	Genesis090	Group2	Unknown	84	D	MAT1-2
AS18170	Mallee Survey Paddock 15	VIC	2018	Genesis090	Group3	Unknown	172	D	MAT1-2
F18090-1	Port Broughton	SA	2018	Genesis090	Group2	Unknown	2	D	MAT1-2
F18090-2	Port Broughton	SA	2018	Genesis090	Group2	Unknown	240	C	MAT1-2
F18093-1	Wandeavah	SA	2018	Genesis090	Group0	Unknown	206	D	MAT1-2
F18093-2	Wandeavah	SA	2018	Genesis090	Group0	Unknown	193	C	MAT1-2
F18096-1	Hart	SA	2018	Genesis090	Group3	Unknown	216	C	MAT1-2
F18096-2	Hart	SA	2018	Genesis090	Group3	Unknown	239	C	MAT1-2
F18112-1	Snowtown	SA	2018	Genesis090	Group3	Unknown	183	D	MAT1-2
F18112-2	Snowtown	SA	2018	Genesis090	Group4	Unknown	168	D	MAT1-2
F18113-1	Port Broughton	SA	2018	Genesis090	Group0	Unknown	213	C	MAT1-2
F18113-2	Port Broughton	SA	2018	Genesis090	Group0	Unknown	238	C	MAT1-2
F18116-1	Port Broughton	SA	2018	Genesis090	Group3	Unknown	245	A	MAT1-2
F18116-2	Port Broughton	SA	2018	Genesis090	Group3	Unknown	182	A	MAT1-2
F18126-1	Maitland	SA	2018	Genesis090	Group4	Unknown	216	C	MAT1-2
F18126-2	Maitland	SA	2018	Genesis090	Group3	Unknown	193	C	MAT1-2
F18149-1	Paskeville	SA	2018	Genesis090	Group3	Unknown	216	C	MAT1-2
F18150-1	Paskeville	SA	2018	Genesis090	Group3	Unknown	216	C	MAT1-2
F18151-1	Paskeville	SA	2018	Genesis090	Group3	Unknown	216	C	MAT1-2
F18165-1	Kilkerrin YP	SA	2018	Genesis090	Group4	Unknown	188	B	MAT1-2
F18165-2	Kilkerrin YP	SA	2018	Genesis090	Group4	Unknown	269	B	MAT1-2
F18166-1	Balaklava	SA	2018	Genesis090	Group3	Unknown	24	A	MAT1-2
TR10329	Westmar	QLD	2018	PBA HatTrick	Group1	Unknown	13	A	MAT1-2
TR10337	GRDC Farm, Tosari, Pampas Kalyx trial site	NSW	2018	Kyabra	Group0	Unknown	241	A	MAT1-2
TR10340	GRDC Farm, Tosari, Pampas Kalyx trial site	NSW	2018	Kyabra	Group0	Unknown	15	B	MAT1-2

Id	Location	State	Year	Host	Patho.Group	Haplotype	MLG	Cluster	Mat.Type
13MAR002	Marnoo	VIC	2013	PBA Slasher	Group0	Unknown	126	B	MAT1-2
13MUR002	Murtoa	VIC	2013	PBA Slasher	Group2	ARH01	249	A	MAT1-2
13RUP002	Rupanyup	VIC	2013	Genesis090	Group2	Unknown	254	C	MAT1-2
FT13092-1	Kingsford	SA	2013	Genesis090	Group4	ARH11	148	E	MAT1-2
FT13092-2	Kingsford	SA	2013	Genesis090	Group4	ARH04	71	E	MAT1-2
FT13092-3	Kingsford	SA	2013	Genesis090	Group1	Unknown	264	B	MAT1-2
FT13092-5	Kingsford	SA	2013	Genesis090	Group1	ARH01	146	B	MAT1-2
FT13093-1	Kingsford	SA	2013	Genesis090	Group1	ARH01	3	E	MAT1-2
FT13093-2	Kingsford	SA	2013	Genesis090	Group1	Unknown	35	E	MAT1-2
FT13093-3	Kingsford	SA	2013	Genesis090	Group1	Unknown	225	E	MAT1-2
FT13093-6	Kingsford	SA	2013	Genesis090	Group0	Unknown	134	A	MAT1-2
FT13095-1	Kingsford	SA	2013	CICA1152	Group0	ARH09	69	E	MAT1-2
TR10345	GRDC Farm, Tosari, Pampas Kalyx trial site	NSW	2018	Kyabra	Group0	Unknown	232	B	MAT1-2
TR10347	GRDC Farm, Tosari, Pampas Kalyx trial site	NSW	2018	Kyabra	Group0	Unknown	252	A	MAT1-2
TR10348	GRDC Farm, Tosari, Pampas Kalyx trial site	NSW	2018	Kyabra	Group0	Unknown	269	B	MAT1-2
TR10350	GRDC Farm, Tosari, Pampas Kalyx trial site	NSW	2018	Kyabra	Group0	Unknown	252	A	MAT1-2
TR10421	Llanver, pdk 17	NSW	2018	PBA HatTrick	Group1	Unknown	167	F	MAT1-2
TR10423	Merrigal	NSW	2018	Almaz	Group0	Unknown	146	B	MAT1-2
TR10424	Merrigal	NSW	2018	Almaz	Group0	Unknown	263	F	MAT1-2
TR10425	Merrigal	NSW	2018	Almaz	Group0	Unknown	112	E	MAT1-2
TR10428	Merrigal	NSW	2018	Almaz	Group1	Unknown	118	A	MAT1-2
TR10430	Merrigal	NSW	2018	Almaz	Group0	Unknown	191	E	MAT1-2
TR10433	Merrigal	NSW	2018	Almaz	Group0	Unknown	269	B	MAT1-2
TR10434	Merrigal	NSW	2018	Almaz	Group0	Unknown	217	B	MAT1-2
TR10435	Merrigal	NSW	2018	Almaz	Group0	Unknown	200	B	MAT1-2
TR10437	Merrigal	NSW	2018	Almaz	Group0	Unknown	269	B	MAT1-2
P2	Unknown	ICARDA	NA	NA	NA	NA	NA	NA	MAT1-1
P4	Kaljebrin	SYRIA (ICARDA)	NA	NA	NA	NA	NA	NA	MAT1-1

Mating Type Assay

Mating types were determined for isolates used in the *A. rabiei* population study. The original DNA samples prepared for submission to Diversity Arrays Sequencing Technologies (DArTseq™) for all Australian isolates were used in the mating type assay. PCR was performed using a multiplex PCR containing MAT-1-1 and MAT1-2-specific forward primers and a common reverse primer, as described in Barve et al. (2003), see Table S2 (inset of Figure S1) for primer details. PCR was performed using standard program (annealing 57℃, 35 cycles) and PCR products were visualised by electrophoresis on 1% agarose gel. Expected band sizes were 702 bp for MAT1-1 and 464 bp for MAT1-2. All Australian isolates have shown the 464 bp band and were designated MAT1-2 (see Figure S1).

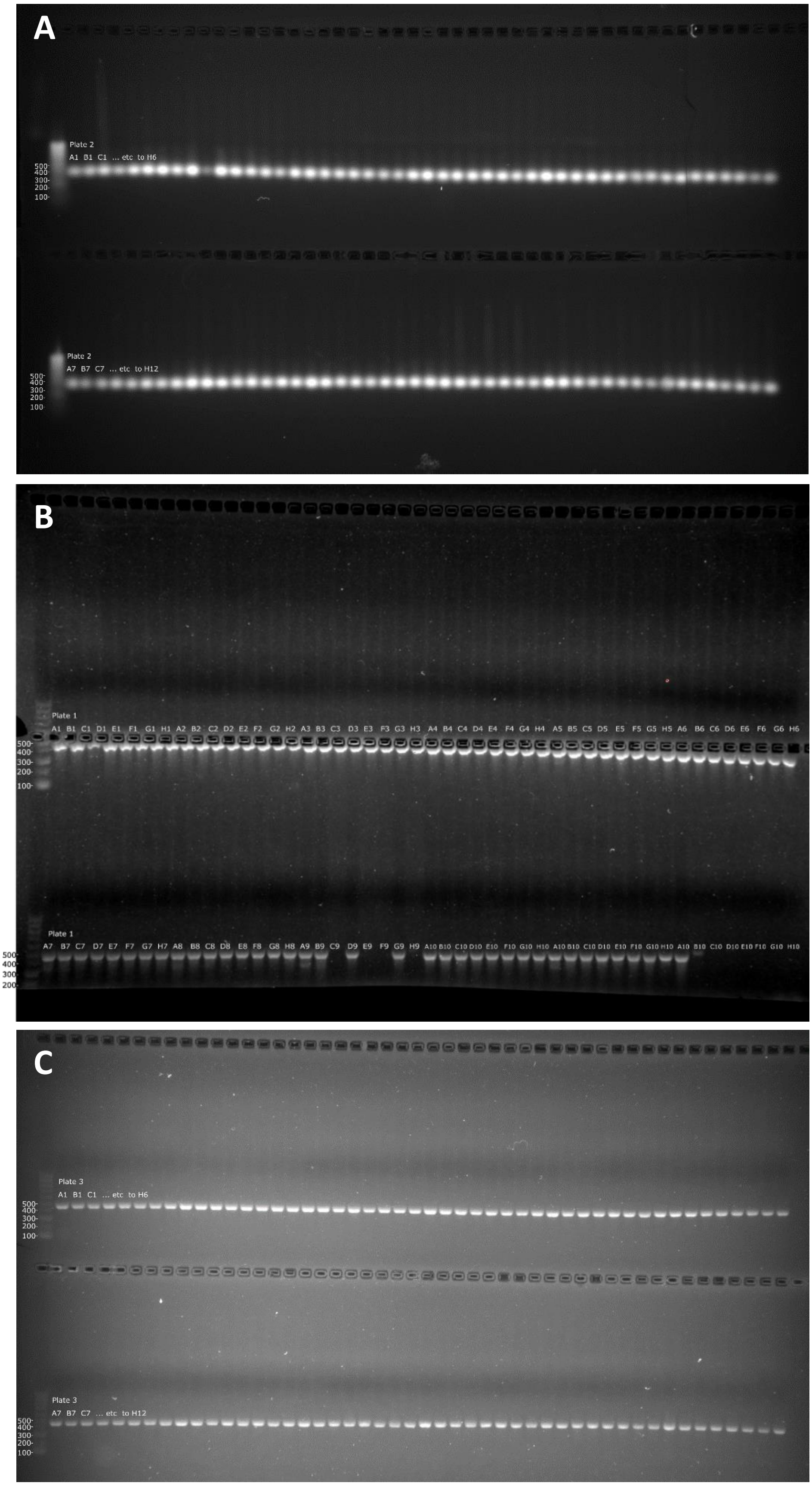

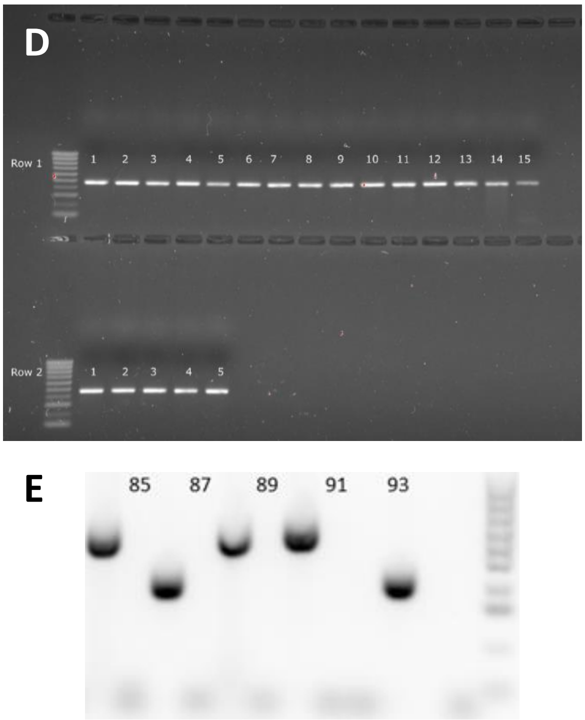

Mating type PCR assay results, as determined by electrophoresis on 1% agarose gel.

Gels **A-D** show the results of the assay with DNA from the Australian *Ascochyta rabiei* isolates as template, which are all consistent with MAT1-2 (464 bp band).

Gel **E** demonstrates the amplification of mating type MAT 1-1 (702 bp) with DNA template from isolates P2 and P4 from ICARDA (in lanes 84 and 88), in addition to MAT 1-1 positive control (lane 90) and 2 verified MAT 1-2 isolates (lanes 86 and 93). Missing bands in gels are empty lanes or no-template controls (water as template).

Table S2. Details of PCR primers used for the mating type assay

SP21_MAT1-1_F	ACAGTGAGCCTGCACAGTTC
Com1_common_R	GCATGCCATATCGCCAGT
Tail5_MAT1-2_F	CGCTATTTTATCCAAGACACACC

Figure S1. Mating type PCR assay results of Australian *Ascochyta rabiei* isolates of this study

Australian *A. rabiei* Population Phylogeny

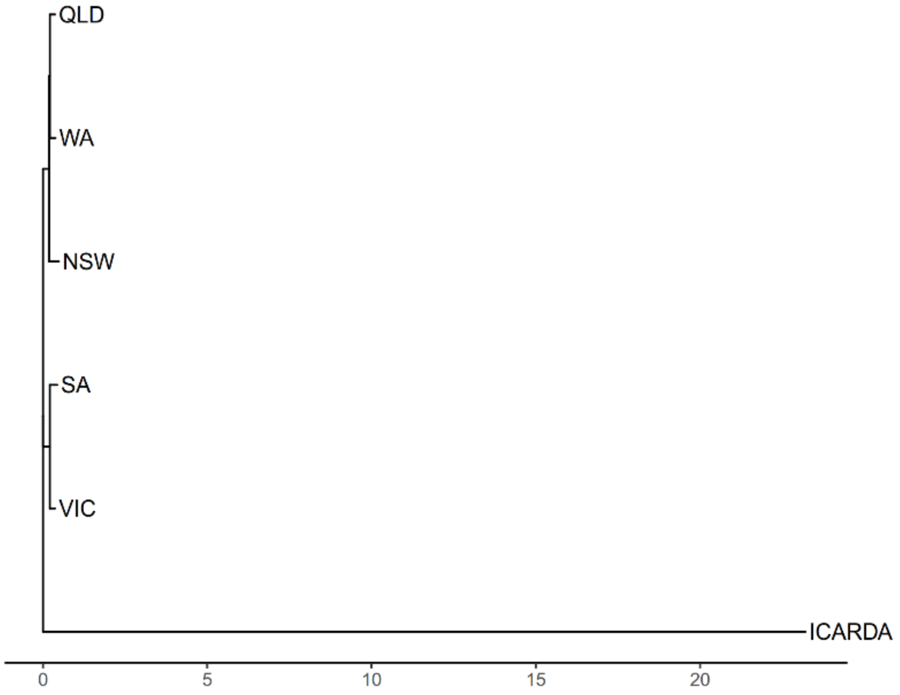

Figure S2. Neighbor-joining phylogenetic tree of Australian *Ascochyta rabiei* populations (isolates from ICARDA provided as an outgroup)
